# Enhanced Thermogenesis in Triple-Negative Breast Cancer Is Associated with Pro-Tumor Immune Microenvironment

**DOI:** 10.3390/cancers13112559

**Published:** 2021-05-23

**Authors:** Shipra Gandhi, Masanori Oshi, Vijayashree Murthy, Elizabeth A. Repasky, Kazuaki Takabe

**Affiliations:** 1 Department of Medical Oncology, Roswell Park Comprehensive Cancer Center, Buffalo, NY 14263, USA; 2 Department of Surgical Oncology, Roswell Park Comprehensive Cancer Center, Buffalo, NY 14263, USA; masanori.oshi@roswellpark.org (M.O.); vijayashree.murthy@roswellpark.org (V.M.); Kazuaki.Takabe@Roswellpark.Org (K.T.); 3 Department of Gastroenterological Surgery, Yokohama City University Graduate School of Medicine, Yokohama, Kanagawa 236-0004, Japan; 4 Department of Immunology, Roswell Park Comprehensive Cancer Center, Buffalo, NY 14263, USA; Elizabeth.repasky@roswellpark.org; 5 Department of Surgery, Niigata University Graduate School of Medical and Dental Sciences, Niigata 951-8510, Japan; 6 Department of Breast Surgery and Oncology, Tokyo Medical University, Tokyo 160-8402, Japan; 7 Department of Surgery, Jacobs School of Medicine and Biomedical Sciences, State University of New York, Buffalo, NY 14263, USA

**Keywords:** thermogenesis, triple-negative breast cancer, cold stress, tumor microenvironment, METABRIC, TCGA, GSE96058

## Abstract

**Simple Summary:**

Preclinical studies have shown that cold stress results in the activation of thermogenesis and an increased tumor growth rate in mice. This study aimed to investigate the clinical relevance of these laboratory findings in patients with triple-negative breast cancer using publicly available large cohorts. Triple-negative breast cancers with high thermogenesis were found to have a pro-tumorigenic immune microenvironment, which may explain the trend towards poor survival observed in this group. This study investigated thermogenesis as a biomarker to predict clinical outcomes, and may pave the way to test novel therapeutics to improve the outcomes of this breast cancer subtype.

**Abstract:**

Mild cold stress induced by housing mice with a 4T1 triple-negative breast cancer (TNBC) cell implantation model at 22 °C increases tumor growth rate with a pro-tumorigenic immune microenvironment (lower CD8 ^+^T cells, higher myeloid-derived suppressor cells (MDSCs) and regulatory T-cells (Tregs)). Since cold stress also activates thermogenesis, we hypothesized that enhanced thermogenesis is associated with more aggressive cancer biology and unfavorable tumor microenvironment (TME) in TNBC patients. A total of 6479 breast cancer patients from METABRIC, TCGA, GSE96058, GSE20194, and GSE25066 cohorts were analyzed using Kyoto Encyclopedia of Genes and Genomes (KEGG) thermogenesis score. High-thermogenesis TNBC was associated with a trend towards worse survival and with angiogenesis, adipogenesis, and fatty acid metabolism pathways. On the other hand, low-thermogenesis TNBC enriched most of the hallmark cell-proliferation-related gene sets (i.e., mitotic spindle, E2F targets, G2M checkpoint, MYC targets), as well as immune-related gene sets (i.e., IFN-α and IFN-γ response). Favorable cytotoxic T-cell-attracting chemokines *CCL5*, *CXCL9*, *CXCL10*, and *CXCL11* were lower; while the MDSC- and Treg-attracting chemokine *CXCL12* was higher. There were higher M2 but lower M1 macrophages and Tregs. In conclusion, high-thermogenesis TNBC is associated with pro-tumor immune microenvironment and may serve as biomarker for testing strategies to overcome this immunosuppression.

## 1. Introduction

Thermogenesis is essential for all animals, ensuring a stable temperature for cellular and physiological functions under conditions of environmental challenge. When faced with the demand to thrive in colder temperatures, human beings increase their body temperature via shivering or non-shivering thermogenesis [1,2]. In shivering thermogenesis, the energy released from muscle contractions produces heat, while brown adipocytes mainly produce heat in non-shivering thermogenesis. The sympathetic nervous system primarily controls thermogenesis in brown and beige adipose tissue by releasing norepinephrine in response to cold stimuli. Brown adipocytes express uncoupling protein 1 (UCP1). The association between complex V and the electron transport chain (ETC) is uncoupled by UCP1, a mitochondrial carrier protein, resulting in dissipation of the proton gradient across the inner mitochondrial membrane. Via this uncoupling, heat is generated instead of ATP [3]. Mice housed at 6 °C, which is 16 °C lower than standard housing temperature, have enhanced thermogenic gene expression with increased production of UCP1 [4]. Pharmacological activation of the beta-3 adrenergic receptor in mice mimics cold exposure, resulting in the activation of thermogenesis [5]. Sympathetic denervation of the adipose tissue inhibits the cold-induced UCP1 expression [4].

The concept that cold stress can induce carcinogenesis has been reported in human epidemiology studies, where cooler environments were associated with greater cancer incidence in a female population in the United States [6]. A negative correlation has been observed between the average annual temperature and incidence rate for 13 types of anatomical site-specific malignancies (uterine, breast, ovary, bladder, melanoma, thyroid, leukemia, etc.) among females [6]. We have shown in a 4T1 murine triple-negative breast cancer (TNBC) cell implantation model that the standard housing temperature required for laboratory mice (22 °C) causes mild chronic cold stress with release of epinephrine and norepinephrine, activating thermogenesis to maintain normal body temperature [7,8]. When this stress is alleviated by housing the mice under thermoneutral temperature at 30 °C, a marked reduction in tumor growth and metastasis is observed. This suppression of tumor growth is dependent upon an effective adaptive immune system at thermoneutrality [8,9]. At the same time, it was also observed that tumor-bearing mice selected a higher ambient temperature than non-tumor-bearing mice. This finding suggested that tumor-bearing mice experience a greater degree of cold stress than non-tumor-bearing mice [8]. Therefore, cold stress is associated with greater cancer incidence in humans [6], and increased tumor growth rate in the 4T1 murine TNBC model [8].

In the past few years, there has been rapid development in microarray and sequencing technologies, which has fundamentally transformed the collection of DNA and RNA data, making them vital for biomedical research. However, the reproducibility across independent studies is limited since the findings are based on gene-level expression [10]. At the same time, interpreting the biological meaning of changes in the expression of a single gene is challenging. The single sample gene set enrichment analysis (ssGSEA) method is an extension of the GSEA that utilizes a single sample instead of a population sample as in the original GSEA application. The degree to which the input gene signature is coordinately upregulated or downregulated within a sample is reflected by the score derived from ssGSEA [11]. The use of a pathway approach reflects gene coordination more accurately, is able to reduce model complexity, and carries the potential to increase the applicability of prediction models [12,13,14,15,16]. Taken together with the fact that thermogenesis should occur under a cold stress environment, we hypothesized that human TNBC with enhanced thermogenesis is associated with aggressive cancer biology and unfavorable tumor immune microenvironment. To test our hypothesis, we analyzed the transcriptome of multiple large human breast cancer cohorts using the Kyoto Encyclopedia of Genes and Genomes (KEGG) thermogenesis score.

## 2. Materials and Methods

### 2.1. Breast Cancer Cohorts

A total of 6479 breast cancer patients were analyzed in this study. Transcriptome and clinical data of 1903 breast cancer patients in the Molecular Taxonomy of Breast Cancer International Consortium (METABRIC) cohort [17] were obtained from cBioPortal [18]; among them, 298 patients had a pathological diagnosis of TNBC. RNA-sequencing data and clinical information of 1065 patients in The Cancer Genome Atlas Breast Cancer Cohort (TCGA-BRCA), among whom 153 patients were diagnosed as having TNBC, were obtained from Pan-Cancer Clinical Data Resource [19] and using the cBio Cancer Genomic Portal [18]. The GSE96058 cohort has the transcriptome profiling of 3273 resected breast cancers from an ongoing study. The latest publicly available clinical data of these patients were obtained from resources noted in a recent The Sweden Cancerome Analysis Network-Breast (SCAN-B) study [20], with 143 TNBC cases. The published data of Shi et al. (GSE20194, *n* = 68) [21] and Symmans et al. (GSE25066, *n* = 170) [22] were used to investigate the association of the thermogenesis scores with treatment response to neoadjuvant chemotherapy (NAC). The analysis shown in this paper was performed within each cohort, and normalization within each cohort was conducted at the time of original publication [17,19,20,21,22]. Disease-free survival (DFS) was defined as the time from primary treatment completion until clinical confirmation of disease recurrence or death. Overall survival (OS) was defined as the time from completion of primary treatment until death. Disease-specific survival (DSS) was defined as the time from completion of primary treatment until death, and deaths due to causes other than the disease were excluded. Patients were classified into two groups based on KEGG thermogenesis pathway. The KEGG thermogenesis pathway gene set was utilized as the score for the analyses in a similar fashion as we have previously published [23]. The top tertile (33%) was defined as “high” thermogenesis score and the bottom two-thirds as “low” thermogenesis score using ssGSEA. ssGSEA calculates a separate enrichment score for each pairing of sample and gene set, independent of phenotype leveling. This transformation allows researchers to characterize cell state in terms of the activity levels of biological processes and pathways rather than through the expression of individual genes. ssGSEA projection transforms the data to a higher-level (pathways instead of genes) space representing a more biologically interpretable set of features on which analytic methods can be applied (ssGSEA v10.0.2, gsea-msigdb.github.io). This study was exempt from Institutional Review Board approval because the information available within the METABRIC, TCGA, GSE96058, GSE20194, and GSE25066 cohorts is publicly accessible and has been de-identified.

### 2.2. Gene Set Enrichment Analysis (GSEA)

Gene set enrichment analysis (GSEA) was performed utilizing the publicly available software provided by the Broad Institute [24], as we have described previously [25,26,27,28,29,30,31,32]. Multiple hypothesis testing for the evaluation of multiple gene sets was corrected using false discovery rate (FDR), where an FDR less than 0.25 was used to define statistical significance, as recommended by the Broad Institute [24]. The hallmark gene set collection of The Molecular Signatures Database (MSigDB) [33] was used for this study, as we described previously [28,29,34,35,36,37,38,39,40,41,42].

### 2.3. Analyses of Infiltrating Immune Cells Using xCell Algorithm

The xCell algorithm [43] was utilized to calculate the fraction of immune cells in the tumor microenvironment through the use of transcriptomic data. The xCell website (https://xcell.ucsf.edu/; accessed on 15 May 2020) was used to obtain the xCell data, as we have reported previously [44,45,46,47,48,49,50,51,52]. The amount of adipocytes was estimated utilizing xCell, as we have reported previously [50], defining adipocytes by the expression pattern of 21 genes, as reported by Aran et al. in the journal *Genome Biology* (2017) (Table S1) [43]. Cytolytic activity ‘CYT’ was calculated using the expression of granzyme A (*GZMA*) and perforin (*PRF1*) as described by Rooney et al. [53]. CYT was analyzed as has been described in our previous work [41,53,54,55,56,57,58,59,60].

### 2.4. Signaling Signature

Interferon (IFN)-γ response and inflammatory signature were calculated using gene set variation analysis (GSVA) [12] with MSigDB hallmark gene sets in two cohorts, METABRIC and GSE96058, as we previously reported [23,29,36,37,38,39,40,51]. Hundreds of genes related to a pathway were analyzed by GSVA (where the coordination of genes was taken into account) as a score that estimates the degree of activation of the pathway. Table S2 lists the 200 genes included in the hallmark inflammatory response gene set as the inflammatory pathway score, which demonstrated that the inflammatory score was significantly associated with better survival and a high level of several immune-related functions in TNBC [38]. The cohorts were divided into high- and low-score groups by median value, as we have previously reported [38].

### 2.5. Statistical Analysis

Other score values of the samples in the TCGA cohort, including the intratumoral heterogeneity, single-nucleotide variant (SNV) neoantigens, indel neoantigens, silent mutation, non-silent mutation, and fraction altered, were calculated and published by Thorsson et al. [61]. Group comparison was calculated using one-way ANOVA test. The Kaplan–Meier method was used to obtain survival statistics using log-rank test. Hazard ratios (HRs) and 95% confidence intervals for survival curves were calculated using a Cox proportional hazards model. Based on DFS, with death being a competing risk event, the cumulative incidence of recurrence was calculated. A two-sided *p* < 0.05 was considered as statistically significant in all the analyses. All boxplots are of Tukey type, with the boxes depicting medians and inter-quartile ranges. R software (version 4.0.1, R Project for Statistical Computing) and Microsoft Excel (version 16 for Windows, Redmond, WA, USA) were utilized for data analysis and data plotting.

## 3. Results

### 3.1. High Thermogenesis Score Is Associated with a Trend towards Worse Survival in Triple-Negative Breast Cancer (TNBC)

**Figure 1:**
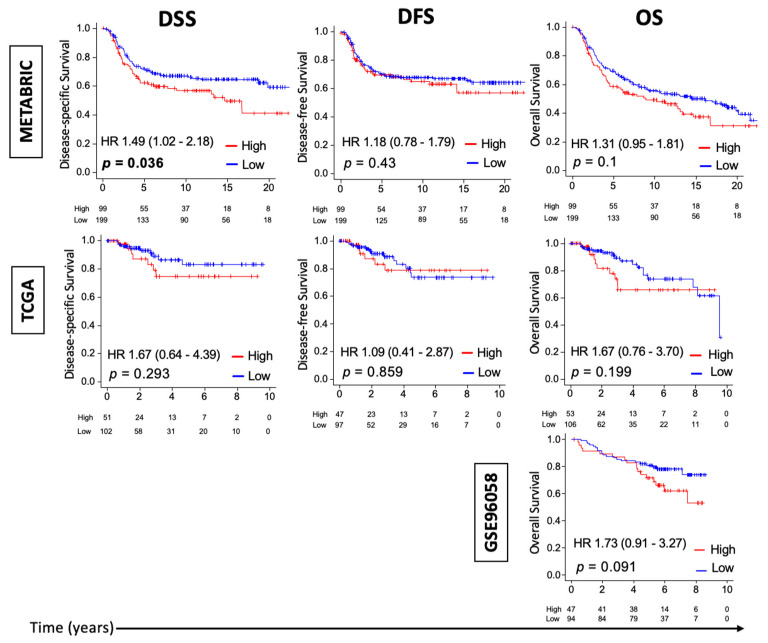
Thermogenesis score and survival outcomes in patients with triple-negative breast cancer (TNBC). Kaplan–Meier survival plots are shown comparing patients with high and low thermogenesis score along with *p*-values and hazard ratios (HRs) with confidence intervals for disease-specific survival (DSS), disease-free survival (DFS), and overall survival (OS) for TNBC. The survival between high- and low-thermogenesis cohorts was compared using log-rank tests.

Given that the preclinical 4T1 murine TNBC cell-line implanted model shows increased tumor growth rate and metastasis during cold stress [8], and because of the literature showing that cold stress results in activation of the thermogenesis pathway [4], we hypothesized that human TNBC with enhanced thermogenesis is associated with increased tumor growth and with poor survival. We examined this in three independent TNBC cohorts: METABRIC (*n* = 298), TCGA (*n* = 153), and GSE96058 (*n* = 141). Thermogenesis score was defined based on KEGG thermogenesis pathway using ssGSEA. Figure S1 shows the KEGG thermogenesis pathway and the list of genes that comprise this thermogenesis pathway. Survival characteristics for TNBC by thermogenesis score in METABRIC, TCGA, and GSE96058 are shown in Figure 1. “High” and “low” thermogenesis scores were defined as the top tertile and bottom two-thirds of each cohort, respectively. High-thermogenesis TNBC had a significantly worse median disease-specific survival (mDSS) compared to low-thermogenesis TNBC in METABRIC (14.7 years vs. not reached and HR = 1.49 (95% C.I. 1.02, 2.18), *p* = 0.036). This survival difference was also observed in TCGA, but was significant only in METABRIC, which has a larger sample size. There was also a trend towards worse DFS in METABRIC and worse OS in all three (METABRIC, TCGA, and GSE96058) for high-thermogenesis TNBC, though these differences were not statistically significant. Thus, our data indicate that TNBC with high thermogenesis score is associated with a statistically significantly worse mDSS or a trend towards worse mDSS. Figure S2 shows the survival characteristics for estrogen receptor (ER)-positive/human epidermal growth factor 2 (Her 2)-negative breast cancer and Her 2-positive breast cancer by thermogenesis score in METABRIC and TCGA cohorts. Similar to TNBC, high-thermogenesis Her 2-positive breast cancer was associated with statistically significantly worse DSS and DFS, but only a trend towards worse overall survival in METABRIC. On the contrary, high-thermogenesis ER-positive/Her 2-negative breast cancer was associated with better survival in METABRIC. However, these results were not validated for either Her 2-positive or ER-positive/Her 2-negative breast cancer in the TCGA cohort. Thus, no clear role of thermogenesis in survival for ER-positive/Her 2-negative or Her 2-positive breast cancer was observed.

### 3.2. Thermogenesis Is Lower in TNBC, and Low-Thermogenesis TNBC Is Associated with Higher Nottingham Histological Grade and Higher MKi67 Expression

**Figure 2:**
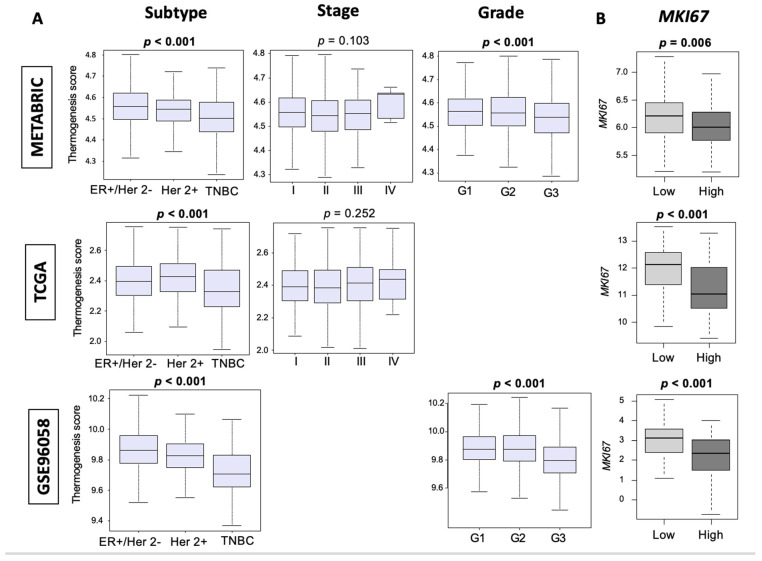
Distribution of thermogenesis score across different subtypes, AJCC stages, and Nottingham histological grades of tumors. (**A**) Tukey boxplots showing the distribution of thermogenesis scores among the three subtypes of breast cancer estrogen receptor (ER)-positive/human epidermal growth factor receptor (Her 2)-negative (ER+/Her 2-), Her 2-positive (Her 2+), and triple-negative breast cancer (TNBC), among four stages (I, II, III, IV) and Nottingham histological grades (G1, G2, G3). Y-axis shows the thermogenesis score. Triple-negative breast cancer and G3 tumors consistently had a lower thermogenesis score with a *p*-value < 0.001 (calculated using one-way ANOVA test). (**B**) Among TNBC, those with lower thermogenesis score were more proliferative with a higher *MKI67* expression. Medians and interquartile ranges are depicted using boxes. One-way ANOVA test was used to calculate the depicted *p*-values.

Given our findings of an increased rate of tumor growth with cold stress in the 4T1 murine TNBC preclinical model and the observed trend towards worse survival in high-thermogenesis TNBC, we expected that high thermogenesis would be associated with aggressive features of cancer including subtypes (ER-positive/Her 2-negative, Her 2-positive and TNBC), American Joint Committee on Cancer (AJCC) cancer staging, Nottingham histological grade (grades 1, 2, and 3), and *MKI67* expression, which is a cell proliferation marker. Although TNBC and grade 3 tumors are known to have worse outcomes and would have been predicted to have high thermogenesis scores, we unexpectedly found that aggressive TNBC subtype and highly proliferative grade 3 tumors both consistently had the lowest thermogenesis score in METABRIC, TCGA, and GSE96058 (Figure 2A, all *p* < 0.001). Since Figure 1 focuses on TNBC and Figure 2 on all the subtypes, it could be speculated that thermogenesis scores may have different relevance for different subtypes of breast cancer (as shown in Figure S2). However, the focus of this paper is on TNBC, given our preclinical model in the TNBC setting, and therefore additional analysis to understand this discrepancy was performed in TNBC. Further, we analyzed the relationship between Ki67 (gene name *MKI67*) and thermogenesis score among TNBC tumors. Interestingly, we observed that high-thermogenesis TNBC was associated with lower *MKI67* expression (Figure 2B, *p* < 0.01). Thus, we found that thermogenesis was low in TNBC and low thermogenesis was associated with higher cell proliferation measured by both pathology (grade) and molecular biology (*MKI67* expression).

### 3.3. High-Thermogenesis TNBC Is Associated with Elevated Intratumoral Adipocytes and the Expression of Mature Vascular-Related Genes, but Not with Mutation Rates

**Figure 3:**
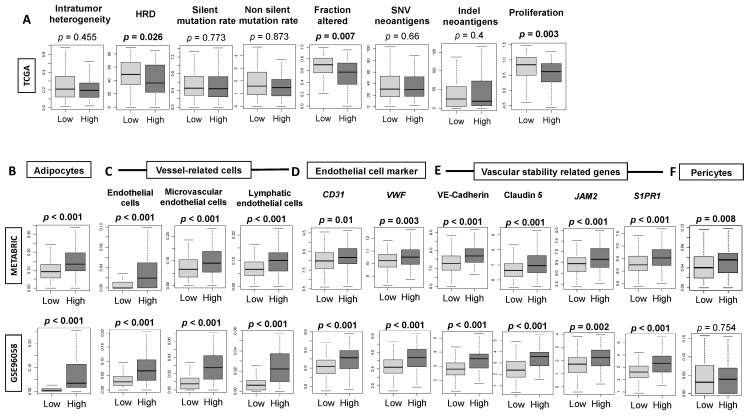
Association between thermogenesis (low/high) and intratumoral heterogeneity, homologous recombination defects (HRDs), mutations, adipocytes, the expression of vessel-related cells, blood endothelial cell markers, and vascular-stability-related genes in the TCGA, METABRIC and GSE96058 cohorts. (**A**) Intratumoral heterogeneity, homologous recombination defects, and mutation-related scores, including mutation rate, fraction altered, single nucleotide variants (SNVs) and indel neoantigens along with proliferation score. (**B**) Abundance of adipocytes in high-thermogenesis TNBC. (**C**) High-thermogenesis TNBC with an abundance of vessel-related cells (endothelial cells, microvascular endothelial cells, and lymphatic endothelial cells). (**D**) High-thermogenesis TNBC showing higher gene expression level of endothelial cell marker-related genes *CD31* (*PECAM1*) and von-Willebrand factor (*vWF*). (**E**) High-thermogenesis TNBC showing higher gene expression levels of vascular-stability-related genes VE-cadherin, Claudin 5, *JAM2*, and sphingosine-1-phosphate-related gene (*S1PR1*). (**F**) Abundance of pericytes. One-way ANOVA test was used to calculate *p*-values.

In order to explain the clinical outcomes in the high-thermogenesis group, we investigated the relationship between thermogenesis and intratumoral heterogeneity, homologous recombination defects (HRDs), mutation rate, and neoantigens, as calculated by Thorsson et al. [62]. We found that high-thermogenesis TNBC had elevated HRDs and fraction altered (Figure 3A, both *p* < 0.05), but no significant differences in intratumoral heterogeneity, mutation rate, or neoantigens were observed. In addition, the findings of lower proliferation in high-thermogenesis TNBC (*p* < 0.01) is consistent with the finding of lower *MKI67* in Figure 2B.

Adipocytes and endothelial cells also constitute the tumor microenvironment. It is well known that there is an interaction between cancer-associated adipocytes with cancer cells. Cancer-associated adipocytes secrete inflammatory cytokines such as interleukin (IL)-6 and tumor necrosis factor (TNF)-α, which are known to promote cancer progression by contributing to pro-cancer inflammation [63,64]. Therefore, we examined the relative distribution of adipocytes in high- and low-thermogenesis TNBC and indeed found higher infiltration in high-thermogenesis TNBC (Figure 3B), which supports the literature data suggesting that higher adipocytes are associated with enhanced thermogenesis. Additionally, IL-6 and leptin produced by adipocytes are known to promote angiogenesis, invasion, and metastasis [65,66], and we have previously shown that intratumoral angiogenesis is associated with metastatic recurrence in breast cancer [37]. Therefore, vascular markers in the high- and low-thermogenesis TNBC were investigated. The relative distribution of endothelial cells, microvascular endothelial cells, and lymphatic endothelial cells was significantly elevated in high-thermogenesis TNBC (Figure 3C). Similarly, endothelial-cell surface marker genes von Willebrand factor (*vWF*) and *CD31* (*PECAM1*) were significantly elevated in high-thermogenesis TNBC, indicating that the concentration of blood vessels inside tumors was increased (Figure 3D). Additionally, genes representing vascular stability and maturity—vascular endothelial cadherin (*CDH5*), claudin 5 (*CLDN5*), and junction adhesion molecule 2 (*JAM2*)—were significantly increased in the high-thermogenesis TNBC (Figure 3E). Sphingosine-1-phosphate (S1P) plays a critical role in angiogenesis, as shown by us and others [67,68,69,70,71,72,73,74,75]. We observed that high-thermogenesis TNBC was also associated with high SIP receptor-1 (*S1PR1*) expression, which is another mature blood vessel marker (Figure 3E). Similarly, high-thermogenesis TNBC was also significantly associated with the presence of more pericytes in METABRIC and a trend in this direction was observed in GSE96058 (Figure 3F). These findings show that high-thermogenesis TNBC is associated with an abundance of adipocytes and mature blood vessels.

### 3.4. Thermogenesis Is Associated with a Trend towards Higher Response to Neoadjuvant Chemotherapy (NAC)

**Figure 4:**
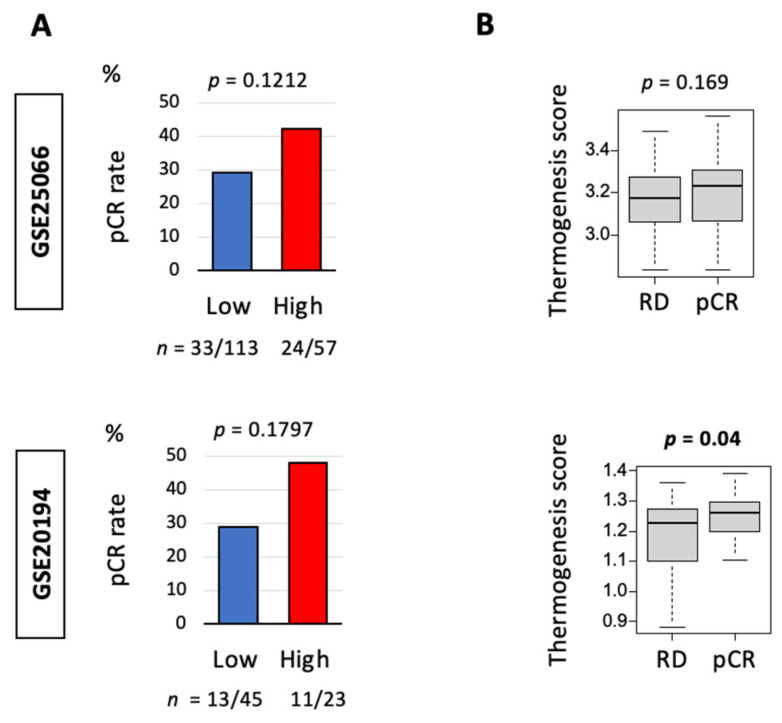
Relationship between thermogenesis and response to neoadjuvant chemotherapy (NAC) in triple-negative breast cancer (TNBC). (**A**) Percentage of pathological complete response (pCR) between low (blue bar) and high (red bar) thermogenesis in GSE25066 (*n* = 170) and GSE20194 (*n* = 68) TNBC cohorts that received NAC. The number of patients where pCR was achieved is shown below the plots. pCR was compared between the two groups using the Fisher’s exact test. (**B**) Tukey boxplots comparing thermogenesis score among patients with pCR or residual disease (RD). Boxplots show median and interquartile level values and Fisher’s exact test was used to calculate the *p*-values.

Given the results showing more aggressive features like higher angiogenesis and the presence of more intratumoral adipocytes in high-thermogenesis TNBC, we expected that the thermogenesis score may be a predictive biomarker for NAC response. This was analyzed using GSE25066 (*n* = 170 TNBC) and GSE20194 (*n* = 70 TNBC) cohorts. As expected, high thermogenesis demonstrated a trend towards higher pathological complete response (pCR) in cohorts GSE25066 and GSE20194, but this was not statistically significant (Figure 4A). At the same time, tumors that attained pCR had statistically significantly higher thermogenesis score compared to tumors with residual disease (RD) after the completion of NAC at the time of surgery in GSE20194, but this result could not be validated in GSE25066 (Figure 4B).

### 3.5. High-Thermogenesis TNBC Enriched Fatty Acid Metabolism and Adipogenesis Pathways while Low-Thermogenesis TNBC Enriched Cell-Proliferation-Related and Immune-Related Pathways

**Figure 5:**
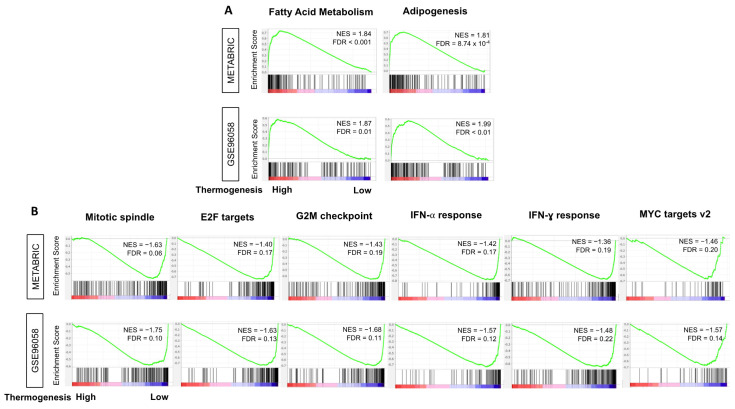
Hallmark gene sets with significant enrichment to high- or low-thermogenesis TNBC in METABRIC and GSE96058 cohorts. Gene set enrichment analysis (GSEA) plots with normalized enrichment score (NES) and false discovery rate (FDR) are shown here for gene sets where significant enrichment was observed in (**A**) high-thermogenesis and (**B**) low-thermogenesis TNBC in METABRIC and GSE96058 cohorts. FDR < 0.25 was used to determine the statistical significance of GSEA. Negative NES refers to enrichment in the low-thermogenesis group.

Given the fact that adipocytes are the main source of heat production during thermogenesis, we expected the fatty acid metabolism and adipogenesis pathways to be enriched in high-thermogenesis TNBC. In order to investigate this, we ran GSEA for the hallmark gene sets in METABRIC and GSE96058 cohorts. As expected, fatty acid metabolism and adipogenesis pathways were significantly enriched in high-thermogenesis TNBC compared to low-thermogenesis TNBC (Figure 5). Since low-thermogenesis TNBC was associated with higher cell proliferation by grade and *MKI67* expression (Figure 2B), it was of interest to identify the hallmark gene sets associated with low thermogenesis. We observed that low-thermogenesis TNBC was consistently enriched in four out of five cell-proliferation-related gene sets, including mitotic spindle, E2F targets, G2M checkpoint, and MYC targets v2, in both cohorts (all FDR < 0.25). At the same time, there was a significant enrichment of immune-related gene sets to low thermogenesis, interferon (IFN)-α response and IFN-γ response, in both cohorts (all FDR < 0.25). Figure S3 shows all the hallmark gene sets with significant enrichment in high- and low-thermogenesis TNBC with FDR < 0.25. The higher enrichment of immune response in low-thermogenesis TNBC leads us to hypothesize that the survival difference by thermogenesis could be partly because low thermogenesis is associated with a favorable tumor immune microenvironment, and thus better survival. Table S3 lists the genes included in the hallmark gene sets with significant enrichment.

### 3.6. Low-Thermogenesis TNBC Is Associated with Higher Cytolytic T-cell (CTL)-Attracting Chemokines, whereas High-Thermogenesis TNBC Is Associated with Higher Myeloid-Derived Suppressor Cell (MDSC)-/Regulatory T-cells (Tregs)-Attracting Chemokines, with Glucocorticoid Receptor (GR) Signaling

**Figure 6:**
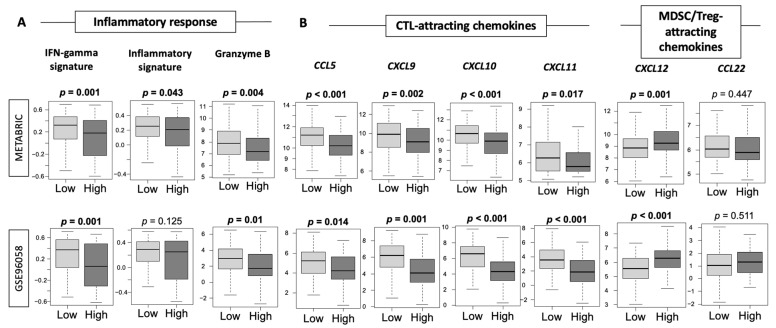

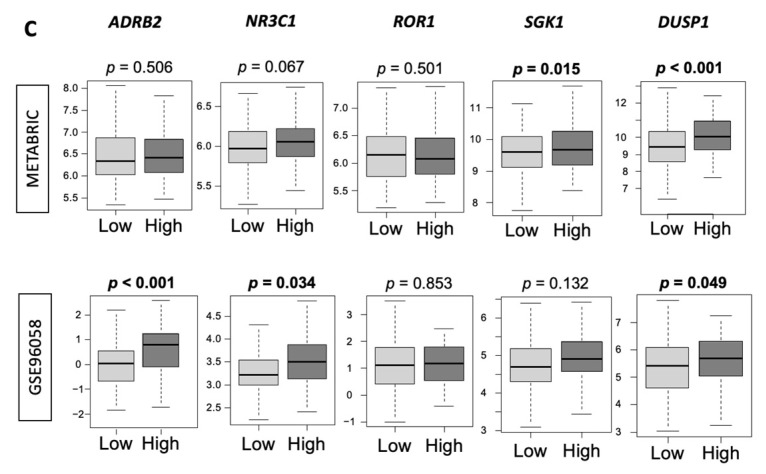
Tukey boxplots of (**A**) inflammatory response, (**B**) chemokines, and (**C**) stress biomarkers using METABRIC and GSE96058 cohorts in TNBC subtype. Y-axis shows the score for respective inflammatory signature/chemokines/stress biomarkers in TNBC tumors with high and low thermogenesis scores (shown on the X-axis). Medians and interquartile ranges are depicted using boxes. One-way ANOVA test was used to calculate the depicted *p*-values.

Given our findings that high-thermogenesis TNBC had more adipocytes and angiogenesis, but lower cancer cell proliferation, which were uniformly shown with multiple methods, we hypothesized that this may be because low-thermogenesis TNBC is associated with a more favorable anti-tumor immune microenvironment, explaining the difference in observed survival outcomes. In order to test this hypothesis, we analyzed the inflammatory signature, as well as chemokines attracting the CTLs (namely CCL5, CXCL9, CXCL10, CXCL11 [76]) and chemokines attracting immunosuppressive MDSCs and Tregs (namely CXCL12 and CCL22 [77,78]) using METABRIC and GSE96058 cohorts. We observed that IFN-γ signature and granzyme B expression were consistently significantly higher in the low-thermogenesis TNBC compared to the high-thermogenesis TNBC in both METABRIC and GSE96058 cohorts (Figure 6A, all *p* < 0.05). Low-thermogenesis TNBC had a significantly higher inflammation signature compared to the high group (*p* < 0.05) in METABRIC and a trend towards a higher inflammatory signature in GSE96058. Tumor infiltration with effector CD8^+^ T cells has been associated with good prognosis in breast cancer [79]; therefore, CTL-attracting chemokines were analyzed. Low-thermogenesis TNBC consistently had a higher expression of the CTL-attracting chemokines *CCL5*, *CXCL9*, *CXCL10*, and *CXCL11* (Figure 6B, all *p* < 0.01). On the other hand, since MDSCs and Tregs are both known to protect tumors from CTL-mediated elimination, and to promote tumor growth, we also analyzed the expression of chemokines attracting MDSCs and Tregs to tumors, that is, *CXCL12* and *CCL22*. As expected, we observed that high-thermogenesis TNBC had a higher expression of *CXCL12* (*p* < 0.01), while no difference in *CCL22* expression was observed. Therefore, we observed that high-thermogenesis TNBC was associated with unfavorable chemokines, whereas low-thermogenesis TNBC was associated with favorable chemokines and inflammation, which could explain the difference in survival. This is consistent with our previous finding that a high inflammatory score in TNBC was significantly associated with better survival and a high level of several immune-related gene sets in GSEA [38]. We showed that although inflammation could be associated with both anti-cancer and pro-cancer immune response, in TNBC, it was associated with favorable anti-cancerous immune response and immune-cell infiltration [38].

Since stress (specifically cold stress) results in higher thermogenesis, we also hypothesized that high-thermogenesis TNBC is associated with a higher expression of stress biomarkers. Stress response is comprised of the autonomic nervous system and the hypothalamic–pituitary–adrenal (HPA) axis. We used markers of both the autonomic nervous system via the beta-adrenergic signaling axis (*ADRB2*) and the HPA axis via glucocorticoid receptor (GR) signaling (*NR3C1*) and the genes downstream of the GR signaling pathway (*ROR1*, *SGK1*, and *DUSP1*) (Figure 6C). No consistent differences in the expression of *ADRB2*, *NR3C1*, *ROR1*, or *SGK1* were observed. Strikingly, high-thermogenesis TNBC was associated with high levels of *DUSP1*, which is downstream of HPA axis activation and GR signaling. Hence, these tumors are GR-driven. GR-driven TNBC has been shown to be associated with worse survival [80].

### 3.7. High-Thermogenesis TNBC Has Lower Anti-Cancer Immune Cell Infiltration, Lower Cytolytic Activity, and Higher M2 Macrophages

**Figure 7:**
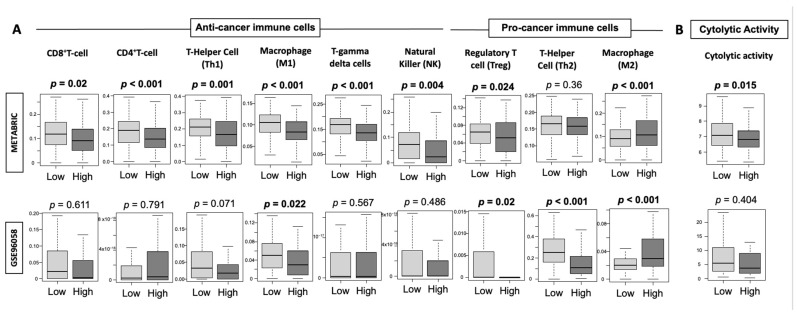
Tukey boxplots of (**A**) anti-cancer and pro-cancer immune cells and (**B**) cytolytic activity in high- and low-thermogenesis TNBC using the xCell algorithm in METABRIC and GSE96058 cohorts. The Y-axis depicts the cellular fraction with low or high thermogenesis expression. Medians and interquartile ranges are shown using boxes. One-way ANOVA test was used to calculate the depicted *p*-values.

Since we observed lower IFN-γ signature and granzyme B expression with lower CTL-attracting chemokines in the high-thermogenesis TNBC, we hypothesized that anti-cancer immune cell infiltration is lower in high-thermogenesis TNBC, and tested this hypothesis in METABRIC and GSE96058 cohorts using the xCell algorithm. Immune-cell fractions, including anti-cancer and pro-cancer immune cells, were compared between the high- and low-thermogenesis groups (Figure 7A). We observed that all the examined anti-cancer immune cells were lower in the high-thermogenesis TNBC group, which may explain the lower inflammatory signature observed in this group. M1 macrophages were consistently significantly lower (*p* < 0.01) in high-thermogenesis TNBC in both cohorts. M1 macrophages secrete pro-inflammatory cytokines, namely, TNF-α, IL-12, CXCL10, and IFN-γ [81], and thus explain the lower IFN-γ and CTL-attracting chemokine CXCL10 expression in high-thermogenesis TNBC. Pro-cancer Tregs followed the same trend as CD8^+^ T cells, which is in agreement with our previous observation [45]. M2 macrophages, which exert an immunosuppressive phenotype by the secretion of anti-inflammatory cytokines (i.e., IL-4, IL-10, and IL-13) [81], were the only pro-cancer immune cells that were higher in high-thermogenesis TNBC. High-thermogenesis TNBC was associated with lower cytolytic activity (CYT, *p* < 0.05), which represents overall ability to kill tumor cells in the tumor microenvironment in METABRIC (Figure 7B). Strikingly consistent results with a trend towards lower CYT in high-thermogenesis TNBC was observed in GSE96058 (Figure 7B).

## 4. Discussion

In this study, we examined the association between thermogenesis score and clinical outcomes, treatment response, and its impact on the tumor microenvironment using bulk tumor transcriptomes from multiple independent cohorts of breast cancer patients. Our study indicates that a high thermogenesis score is associated with a trend towards worse survival outcomes in TNBC. As expected, a higher thermogenesis score was able to predict better outcomes to neoadjuvant chemotherapy given its more aggressive cancer biology. High-thermogenesis TNBC was enriched in pathways for fatty acid metabolism and adipogenesis, with more angiogenesis gene expression and lower CTL-attracting chemokines and higher MDSC-/Treg-attracting chemokine. On the other hand, low-thermogenesis TNBC had higher cell proliferation, which could be explained by greater cytolytic activity and enrichment for genes for IFN response compared to high-thermogenesis TNBC.

The KEGG thermogenesis pathway gene set was used to divide the tumors into high and low thermogenesis score groups. The KEGG is a database resource for understanding the high-level functions and utilities of biological systems such as cells, organisms, and ecosystems, from genomic and molecular-level information. It is a computer representation of a biological system, consisting of molecular building blocks of genes and proteins and chemical substances, integrated with the knowledge on molecular wiring diagrams of interaction, reaction, and relation networks. It also contains disease and drug information as perturbations to the biological system. The KEGG database has been in development by Kanehisa Laboratories since 1995, and is now a prominent reference knowledge base for the integration and interpretation of large-scale molecular datasets generated by genome sequencing and other high-throughput experimental technologies. The KEGG gene sets are widely accepted and recognized to represent specific and well-defined biological statuses or processes, and to display coherent expression [82,83,84]. The thermogenesis score was calculated by ssGSEA algorithm using the KEGG thermogenesis gene sets.

The finding that high-thermogenesis TNBC has a trend towards worse survival but responds well to NAC may seem paradoxical, though it is important to note that our study did not demonstrate statistical significance for any of these results. Hence, the discussion is hypothesis-generating. There are several possible explanations for this observed discrepancy. Firstly, it is important to note that a biomarker may associate with higher chemosensitivity but also with worse survival when it is associated with aggressive biology. An example is our recently published study where we demonstrated that the 4-gene score was associated with poor survival and aggressive clinical parameters in patients with breast cancer, but the score was also associated with a higher rate of pCR [28]. Another example is higher androgen receptor expression in ER-positive breast cancer, where higher expression is associated with worse response to NAC but better survival [27]. Therefore, with respect to the aggressive nature of the high-thermogenesis group, it is expected that this group will overall have poor survival outcomes. Secondly, although pCR is often used as a surrogate for improved survival outcomes [85], it is important to note that pCR improvement does not always translate into improved DFS, as observed in CALGB 40603 when carboplatin addition to chemotherapy resulted in improved pCR but not in DFS [86]. There are multiple similar examples where improvement in pCR with certain treatments did not translate into a survival benefit [85], such as the addition of lapatinib to trastuzumab in the ALTTO trial [87], or the addition of bevacizumab to chemotherapy in the BEATRICE trial [88]. Even though we only observed a trend and were unable to statistically validate the poor outcomes in high-thermogenesis TNBC in this study, our preclinical model in murine TNBC showed an increased rate of tumor growth on exposure to cold stress [8], and epidemiological data also support that cold stress may correlate with higher cancer incidence [6]. Therefore, it was of interest to investigate the tumor immune microenvironment to understand the immune cell infiltration that is associated with these differences.

Studies have shown that thermal stress (cold stress) induced by housing 4T1 tumor-bearing mice at 22 °C compared to 30 °C decreases the overall ability of the anti-tumor immune response to control tumor growth by decreasing the frequency and function of CD8^+^ T cells and promoting the suppressive function of MDSCs [89], and increasing Tregs [9]. This change in the tumor microenvironment explains the weaker anti-cancer immune response during thermal stress. Our observation of lower IFN-γ and granzyme B expression in high-thermogenesis TNBC along with lower CYT is consistent with these known findings showing lower anti-cancer activity of CD8^+^ T cells in TNBC with high thermogenesis, and, hence, thermal stress. MDSCs are well-known to reduce CD8^+^ T-cell proliferation and IFN-γ production [90], and therefore our results also support that high-thermogenesis TNBC would indirectly have higher MDSCs compared to low-thermogenesis TNBC. In support of the above findings, our study showed that high-thermogenesis TNBC had significantly lower expression of CTL-attracting chemokines such as *CCL5*, *CXCL9*, *CXCL10*, and *CXCL11* and higher expression of the MDSC- and Treg-attracting chemokine *CXCL12,* which also explains the lower cytolytic activity in high-thermogenesis TNBC, reflecting thermal stress. This could imply that this group of patients with TNBC experiencing thermal stress may represent “cold” tumors that may derive minimal benefit from immunotherapeutic strategies and would need novel agents to enhance immunotherapy efficacy. Interestingly, we observed that *DUSP1* expression was higher in high-thermogenesis TNBC. *DUSP1* is a downstream gene upregulated upon GR activation. Activation of the GR pathway has been shown to be associated with worse survival in TNBC [80]. The higher *DUSP1* expression in the high-thermogenesis TNBC could lead us to hypothesize that stress pathways activating GR signaling are also enhanced in the high-thermogenesis group, which may contribute to the observed outcomes.

There is a complex interplay between thermogenesis and angiogenesis. It is well-known that the IL-6 and leptin produced by adipocytes promote angiogenesis [65,66]. Adipose tissue (especially brown adipose tissue) is highly vascularized. This vasculature plays an important role in supplying nutrients and oxygen to adipocytes, removing metabolic products, and conducting the heat produced in adipose tissue to the rest of the body. The vessel wall itself also serves as a source of stem cells to later differentiate into preadipocytes or adipocytes. In fact, vascular endothelial cells and adipocytes are the two main cellular components in the adipose microenvironment. Adipocytes also produce angiogenic factors like vascular endothelial growth factor (VEGF) that regulate angiogenesis, vascular survival, vascular remodeling, and blood perfusion [91]. Studies have shown that both cold-induced sympathetic activation [92] and beta-3 adrenergic agonist [93] are able to augment adipose angiogenesis during the browning of white adipose tissue. This results in the upregulation of UCP1, which is required for non-shivering thermogenesis. These findings are consistent with our study showing that high-thermogenesis TNBC is associated with a higher expression of mature vascular-related genes, that is, higher angiogenesis.

It is well known that beta-adrenergic signaling promotes invasion, epithelial-to-mesenchymal transition phenotype [94], and the generation of an immunosuppressive tumor microenvironment [9], although we did not find any difference in *ADBR2* (receptor for beta2-adrenergic signaling) gene expression between high- and low-thermogenesis TNBC in our study. Cold stress is one among many stressors that is commonly experienced by humans (others include psychological or emotional stress) which could be significantly increased with a diagnosis of cancer. This is mediated through the sympathetic nervous system. We have shown in a phase 1 clinical trial that the abrogation of stress in metastatic melanoma patients via pharmacological approaches (e.g., inhibition of the beta-adrenergic signaling axis via the non-selective beta-blocker propranolol) has the potential to improve clinical outcomes [95]. Similarly, in order to abrogate stress in breast cancer, several pharmacological and non-pharmacological strategies are underway in order to improve breast cancer outcomes [96]. Hiller et al. showed that perioperative pharmacological blockade of the beta-adrenergic signaling pathway in breast cancer reduced biomarkers of metastasis and resulted in the creation of a favorable immune profile in breast cancer [97]. Since cold stress has been shown to result in enhanced thermogenesis, we speculate that transcriptomic profiling of tumors to delineate high thermogenesis has the potential to identify tumors that may selectively benefit from pharmacological (beta blockers) or non-pharmacological (yoga, meditation, exercise, natural products, acupuncture, support groups, psychological counselling) stress abrogation strategies. In addition, we also observed that low-thermogenesis TNBC had a higher expression of HRDs. The presence of these aberrant DNA-repair pathways makes the tumor sensitive to DNA damaging therapies such as PARP inhibitors and platinum agents. Low-thermogenesis TNBC was also associated with higher fraction altered/copy number alteration (CNA), which are defined as acquired changes in the copy number of genes in tissues such as tumors. Higher genomic instability and malignant transformation is associated with an increased burden of CNAs [98]. Several novel therapeutics are currently under development and in clinical trials to target this population with defects in HR. Clinical trials using PARP inhibitors for breast cancer with germline *BRCA1/2* mutations have shown promising results in the neoadjuvant [99] and metastatic setting [100,101], and are being actively studied in TNBC with HRDs [102]. Thus, it is possible that low-thermogenesis TNBC with high HRDs may be sensitive to PARP inhibitors and platinum chemotherapy, which would be an interesting area of research to explore further.

Limitations in our study include the fact that is it a retrospective analysis of cohorts, and lacks experimental validation using patient samples since we did not have access to those samples. Therefore, data interpretation is limited due to the lack of a mechanistic approach and causality association. Although the survival data in TNBC is intriguing, it will require validation in other cohorts with large numbers of patients since the trends observed here were not statistically significant. However, the finding of differences in tumor immune microenvironment between high and low thermogenesis in human TNBC provides validation of the cold stress theory emerging from prior preclinical experiments, and is hypothesis-generating. This also supports the observation of higher cancer incidence among females with cancer living in cold environments, which could indirectly imply that an activated thermogenesis pathway results in enhanced tumorigenesis in females and thus is relevant to this study in breast cancer, which is predominantly a female malignancy. Additionally, no clear role of thermogenesis on survival in ER-positive/Her 2-negative and Her 2-positive breast cancer was observed in our study, and should be validated in other larger patient cohorts. Future work in order to further advance this field should aim to understand the interaction of stress response via thermoregulation and antitumor immunity in different subtypes of breast cancer, as well as in other malignancies. This would help to validate high thermogenesis as a biomarker to identify cancers that are stressed in order to utilize both pharmacological and non-pharmacological approaches to abrogate stress in order to improve outcomes. Our ongoing phase 2 clinical trial in metastatic melanoma (ClinicalTrials.gov Identifier: NCT03384836) is investigating a validated stress questionnaire quantifying subjective stress to assess the benefit of pharmacological stress blockade with propranolol for high/moderate/low-stressed melanoma patients.

## 5. Conclusions

We observed that high-thermogenesis TNBC was associated with a trend towards worse survival and with a pro-tumor immune microenvironment. Further research is warranted to validate if high thermogenesis could reflect tumors under thermal or other types of stress and to develop novel strategies to abrogate stress and improve outcomes.

## Data Availability

Publicly available datasets were analyzed in this study. This data can be found here; cBioPortal for Cancer Genomics; and Home—GEO—NCBI (nih.gov).

## Supplementary Materials

The following are available online at https://www.mdpi.com/article/10.3390/cancers13112559/s1. Table S1: The genes that were used to define adipocytes in xCell, a bioinformatics algorithm reported by Aran et al. The algorithm is unable to differentiate between white and brown adipocytes. Table S2: Genes in the hallmark inflammatory response gene set. Table S3: List of genes included in the hallmark gene sets with significant enrichment. Figure S1: (A) Kyoto Encyclopedia of Genes and Genomes (KEGG) thermogenesis pathway. (B) List of genes comprising the KEGG thermogenesis pathway and their description. Figure S2: Thermogenesis score and survival characteristics in patients with (A) estrogen receptor (ER)-positive/human epidermal growth factor receptor 2 (Her 2)-negative breast cancer and (B) Her 2-positive breast cancer. Kaplan–Meier survival plots are shown comparing high and low thermogenesis score along with *p*-values for disease-specific survival (DSS), disease-free survival (DFS), and overall survival (OS). The survivals between high and low thermogenesis groups were compared using log-rank tests. Figure S3: Hallmark gene sets with significant enrichment in triple-negative breast cancer (TNBC) with high and low thermogenesis scores in METABRIC, TCGA, and GSE96058 cohorts. Gene set enrichment analysis (GSEA) plots along with the normalized enrichment score (NES) and false discovery rate (FDR) are shown for the gene sets where enrichment was seen in high-thermogenesis and low-thermogenesis tumors in METABRIC and GSE96058 cohorts. The cut-off of highest tertile (top 33%) of thermogenesis score was considered as high and the remaining as low. FDR < 0.25 was used to determine the statistical significance. Negative NES refers to gene set enrichment in the low thermogenesis group.
