# Supplementary material for: Enhanced Thermogenesis in Triple-Negative Breast Cancer Is Associated with Pro-Tumor Immune Microenvironment
Source: Cancers (Basel). 2021 May 23;13(11):2559. doi: 10.3390/cancers13112559 (PMC8197168; doi:10.3390/cancers13112559)
Supplement: Supplementary file 1 [file cancers-13-02559-s001.zip › cancers-1193828-supplementary.pdf]

Enhanced Thermogenesis in Triple-Negative Breast Cancer Is Associated with Pro-Tumor Immune Microenvironment

Table S1. The genes that were used to define adipocytes in xCell, a bioinformatics algorithm reported by Aran et al. (1). The algorithm is unable to differentiate between white and brown adipocytes.

Genes used to define adipocytes.
ADH1B, ADIPOQ, ATP1A2, ATP5G3, C6, CILP, COL5A3, DBI, DLAT, ECHDC1, GPD1, HADHA, HP, LBP, PLIN1, PNPLA2, PPP1R1A, PPP2R1B, PTGER3, SLC25A6, TF

Table S2. Genes in the hallmark inflammatory response gene set.

HALLMARK_INFLAMMATORY_RESPONSE	
ABCA1	ATP Binding Cassette subfamily A Member 1
ABI1	ABI Interactor 1
ACVR1B	Activin Receptor Type 1B
ACVR2A	Activin Receptor Type 2A
ADGRE1	Adhesion G Protein Coupled Receptor E1
ADM	Adrenomedullin
ADORA2B	Adenosine A2B Receptor
ADRM1	Adhesion Regulating Molecule 1
AHR	Aryl Hydrocarbon Receptor
APLNR	Apelin Receptor
AQP9	Aquaporin-9
ATP2A2	ATPase Sarcoplasmic/Endoplasmic Reticulum Ca2+ Transporting 2
ATP2B1	ATPase Plasma Membrane Ca2+ Transporting 1
ATP2C1	ATPase Secretory Pathway Ca2+ Transporting 1
AXL	AXL Receptor Tyrosine kinase
BDKRB1	Bradykinin Receptor B1
BEST1	Bestrophin-1
BST2	Bone Marrow stromal Cell Antigen 2
BTG2	BTG Anti-Proliferation Factor 2
C3AR1	Complement C3a Receptor 1
C5AR1	Complement C5a Receptor 1
CALCRL	Calcitonin Receptor Like Receptor
CCL17	C-C motif Chemokine Ligand 17
CCL2	C-C Motif Chemokine Ligand 2
CCL20	C-C Motif Chemokine Ligand 20
CCL22	C-C Motif Chemokine Ligand 22
CCL24	C-C Motif Chemokine Ligand 24

CCL5	C-C Motif Chemokine Ligand 5
CCL7	C-C Motif Chemokine Ligand 7
CCR7	C-C Motif Chemokine Receptor 7
CCRL2	C-C Motif Chemokine Receptor Like 2
CD14	Cluster of Differentiation 14
CD40	Cluster of Differentiation 40
CD48	Cluster of Differentiation 48
CD55	Cluster of Differentiation 55
CD69	Cluster of Differentiation 69
CD70	Cluster of Differentiation 70
CD82	Cluster of Differentiation 82
CDKN1A	Cyclin Dependent Kinase Inhibitor 1A
CHST2	Carbohydrate Sulfotransferase 2
CLEC5A	C-type Lectin Domain Family 5 Member A
CMKLR1	Chemokine Like Receptor 1
CSF1	Colony Stimulating Factor 1
CSF3	Colony Stimulating Factor 3
CSF3R	Colony Stimulating Factor 3 Receptor
CX3CL1	C-X3-C Motif Chemokine Ligand 1
CXCL10	C-X-C Motif Chemokine ligand 10
CXCL11	C-X-C Motif Chemokine ligand 11
CXCL6	C-X-C Motif Chemokine ligand 6
CXCL8	C-X-C Motif Chemokine ligand 8
CXCL9	C-X-C Motif Chemokine ligand 9
CXCR6	C-X-C Chemokine Receptor Type 6
CYBB	Cytochrome B-245 Beta Chain
DCBLD2	Discoidin, CUB and LCCL Domain Containing 2
EBI3	Epstein-Barr Virus Induced 3
EDN1	Endothelin 1
EIF2AK2	Eukaryotic Translation Initiation Factor 2 Alpha Kinase 2
EMP3	Epithelial membrane Protein 3
EREG	Epiregulin
F3	Coagulation Factor III
FFAR2	Free fatty Acid Receptor 2
FPR1	Formyl Peptide Receptor 1
FZD5	Frizzled 5
GABBR1	Gamma-Aminobutyric Acid Type B Receptor Subunit 1
GCH1	GTP Cyclohydrolase 1
GNA15	Guanine Nucleotide Binding Protein Subunit Alpha 15
GNAI3	Guanine Nucleotide Binding Protein Subunit Alpha I3
GP1BA	Glycoprotein lb Platelet Subunit Alpha
GPC3	Glypican 3
GPR132	G Protein Coupled Receptor 132

GPR183	G Protein Coupled Receptor 183
HAS2	Hyaluronan Synthase 2
HBEGF	Heparin Binding EGF Like Growth Factor
HIF1A	Hypoxia Inducible Factor 1 Alpha
HPN	Hepsin
HRH1	Histamine H1
ICAM1	Intercellular Adhesion Molecule 1
ICAM4	Intercellular Adhesion Molecule 4
ICOSLG	Inducible T Cell Costimulator Ligand
IFITM1	Interferon Induced Transmembrane Protein 1
IFNAR1	Interferon Alpha and Beta Receptor Subunit 1
IFNGR2	Interferon Gamma Receptor 2
IL10	Interleukin 10
IL10RA	Interleukin 10 Receptor Subunit Alpha
IL12B	Interleukin 12 Subunit Beta
IL15	Interleukin 15
IL15RA	Interleukin 15 Receptor Subunit Alpha
IL18	Interleukin 18
IL18R1	Interleukin 18 Receptor 1
IL18RAP	Interleukin 18 Receptor Accessory Protein
IL1A	Interleukin 1 Alpha
IL1B	Interleukin 1 Beta
IL1R1	Interleukin 1 Receptor 1
IL2RB	Interleukin 2 Receptor Subunit Beta
IL4R	Interleukin 4 Receptor
IL6	Interleukin 6
IL7R	Interleukin 7 Receptor
INHBA	Inhibin Subunit Beta A
IRAK2	Interleukin 1 Receptor Associated Kinase 2
IRF1	Interferon Regulatory Factor 1
IRF7	Interferon Regulatory Factor 7
ITGA5	Integrin Alpha 5
ITGB3	Integrin Subunit Beta 3
ITGB8	Integrin Subunit Beta 8
KCNA3	Potassium Voltage Gated Channel Subfamily A Member 3
KCNJ2	Potassium Inwardly Rectifying Channel Subfamily J Member 2
KCNMB2	Potassium Calcium Activated Channel Subfamily M Regulatory Beta Subunit 2
KIF1B	Kinesin Family Member 1B
KLF6	Krueppel Like Factor 6
LAMP3	Lysosome Associated Membrane Glycoprotein 3
LCK	Lymphocyte Specific Protein Tyrosine Kinase
LCP2	Lymphocyte Cytosolic Protein 2

LDLR	Low Density Lipoprotein Receptor
LIF	Leukemia Inhibitory Factor
LPAR1	Lysophosphatidic Acid Receptor 1
LTA	Lymphotoxin Alpha
LY6E	Lymphocyte Antigen 6E
LYN	LYN Proto Oncogene
MARCO	Macrophage Receptor
MEFV	Mediterranean Fever
MEP1A	Meprin A Subunit Alpha
MET	Mesenchymal-Epithelial Transition Proto Oncogene
MMP14	Matrix Metalloproteinase 14
MSR1	Macrophage Scavenger Receptor 1
MXD1	MAX Dimerization Protein 1
MYC	MYC Proto Oncogene
NAMPT	Nicotinamide Phosphoribosyltransferase
NDP	Norrie Disease Protein
NFKB1	Nuclear factor Kappa B Subunit 1
NFKBIA	NFkB Inhibitor Alpha
NLRP3	NLR Family Pyrin Domain Containing 3
NMI	N Myc and STAT Interactor
NMUR1	Neuromedin U Receptor 1
NOD2	Nucleotide Binding oligomerization Domain Containing Protein 2
NPFFR2	Neuropeptide FF Receptor 2
OLR1	Oxidized Low Density lipoprotein Receptor 1
OPRK1	Opioid Receptor Kappa 1
OSM	Oncostatin M
OSMR	Oncostatin M Receptor
P2RX4	P2X Purinoceptor 4
P2RX7	P2X Purinoceptor 7
P2RY2	P2Y Purinoceptor 2
PCDH7	Protocadherin 7
PDE4B	Phosphodiesterase 4B
PDPN	Podoplanin
PIK3R5	Phosphoinositide 3 Kinase Regulatory Subunit 5
PLAUR	Plasminogen Activator, Urokinase Receptor
PROK2	Prokineticin 2
PSEN1	Presenilin 1
PTAFR	Platelet Activating Factor Receptor
PTGER2	Prostaglandin E Receptor 2
PTGER4	Prostaglandin E Receptor 4
PTGIR	Prostaglandin I2
PTPRE	Protein Tyrosine Phosphatase Receptor Type E
PVR	PVR Cell Adhesion Molecule

RAF1	Raf 1 Proto Oncogene
RASGRP1	RAS Guanyl Releasing Protein 1
RELA	V-rel Avian Reticuloendotheliosis Viral Oncogene Homolog A
RGS1	Regulator of G Protein Signaling 1
RGS16	Regulator of G Protein Signaling 16
RHOG	Ras Homology Growth Related
RIPK2	Receptor interacting Serine and Threonine Protein Kinase 2
RNF144B	Ring Finger Protein 144B
ROS1	ROS Proto Oncogene 1
RTP4	Receptor Transporter Protein 4
SCARF1	Scavenger Receptor Class F Member 2
SCN1B	Sodium Channel Subunit Beta 1
SELE	Selection E
SELENOS	Selenoprotein S
SELL	Selectin L
SEMA4D	Semaphorin 4D
SERPINE1	Serpin Family E Member 1
SGMS2	Sphingomyelin Synthase 2
SLAMF1	Signaling Lymphocytic Activation Molecule 1
SLC11A2	Solute Carrier Family 11 Member 2
SLC1A2	Solute Carrier Family 1 Member 2
SLC28A2	Solute Carrier Family 28 Member 2
SLC31A1	Solute Carrier Family 31 Member 1
SLC31A2	Solute Carrier Family 31 Member 2
SLC4A4	Solute Carrier Family 4 Member 4
SLC7A1	Solute Carrier Family 7 Member 1
SLC7A2	Solute Carrier Family 7 Member 2
SPHK1	Sphingosine Kinase 1
SRI	Sorcin
STAB1	Stabilin 1
TACR1	Tachykinin Receptor 1
TACR3	Tachykinin Receptor 3
TAPBP	TAP Binding Protein
TIMP1	TIMP Metallopeptidase Inhibitor 1
TLR1	Toll Like Receptor 1
TLR2	Toll Like Receptor 2
TLR3	Toll Like Receptor 3
TNFAIP6	Tumor Necrosis Factor Alpha Induced Protein 6
TNFRSF1B	Tumor Necrosis Factor Receptor Superfamily Member 1B
TNFRSF9	Tumor Necrosis Factor Receptor Superfamily Member 9
TNFSF10	Tumor Necrosis Factor Superfamily Member 10
TNFSF15	Tumor Necrosis Factor Superfamily Member 15
TNFSF9	Tumor Necrosis Factor Superfamily Member 9

TPBG	Trophoblast Glycoprotein
VIP	Vasoactive Intestinal Peptide

Figure S1: (A) Kyoto Encyclopedia of Genes and Genomes (KEGG) thermogenesis pathway. (B) List of genes comprising the KEGG thermogenesis pathway and their description.

1(A):

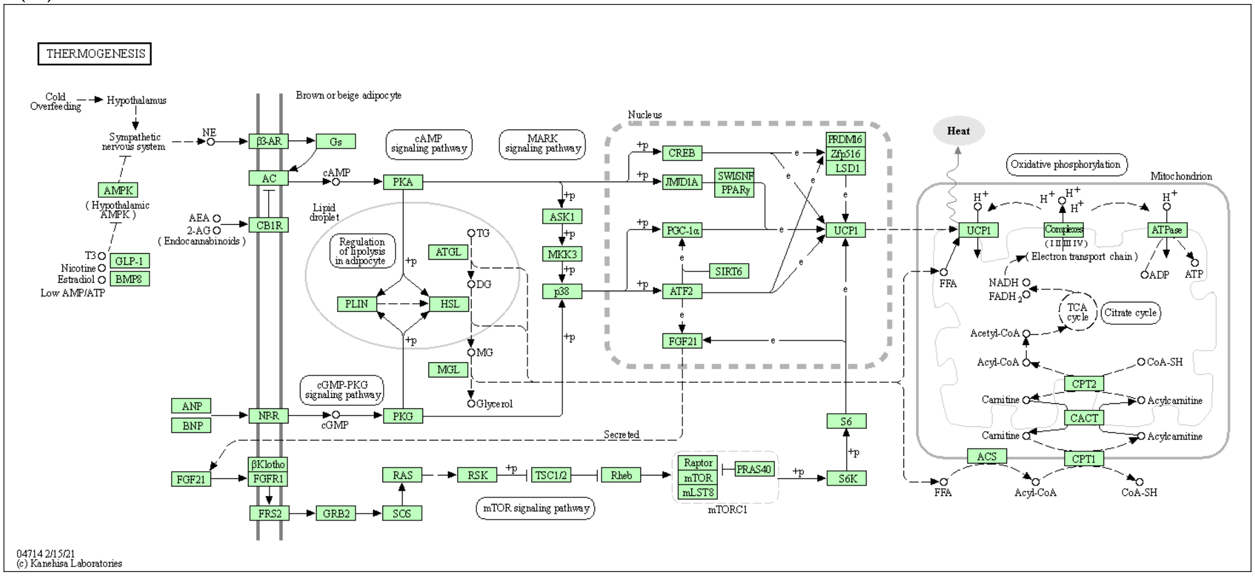

1(B):

No.	Gene Symbol	Gene Name	Function
1	ADRB3	adrenoceptor beta 3	Beta-adrenergic receptors mediate the catecholamine-induced activation of adenylate cyclase through the action of G proteins. Beta-3 is involved in the regulation of lipolysis and thermogenesis.
2	GNAS	GNAS complex locus	Ligand–receptor binding results in detachment of the G protein, switching it to an "on" state and permitting Gα activation of second messenger signaling cascades.
3	ADCY1	adenylate cyclase 1	Catalyzes the formation of the signaling molecule cAMP in response to G-protein signaling. Mediates responses to increased cellular Ca(2+)/calmodulin levels.
4	ADCY2	adenylate cyclase 2	Catalyzes the formation of the signaling molecule cAMP in response to G-protein signaling.
5	ADCY3	adenylate cyclase 3	Catalyzes the formation of the signaling molecule cAMP in response to G-protein signaling. Participates in signaling cascades triggered by odorant receptors via its function in cAMP biosynthesis.
6	ADCY4	adenylate cyclase 4	Catalyzes the formation of the signaling molecule cAMP in response to G-protein signaling.
7	ADCY5	adenylate cyclase 5	Catalyzes the formation of the signaling molecule cAMP in response to G-protein signaling.<br>Mediates signaling downstream of ADRB1.
8	ADCY6	adenylate cyclase 6	Catalyzes the formation of the signaling molecule cAMP downstream of G protein-coupled receptors.<br>Functions in signaling cascades downstream of beta-adrenergic receptors in the heart and in vascular smooth muscle cells.

9	ADCY7	adenylate cyclase 7	Catalyzes the formation of cAMP in response to activation of G protein-coupled receptors (probable). Functions in signaling cascades activated namely by thrombin and sphingosine 1-phosphate and mediates regulation of cAMP synthesis through synergistic action of the stimulatory G alpha protein with GNA13.
10	ADCY8	adenylate cyclase 8	Catalyzes the formation of cAMP in response to calcium entry leadings to cAMP signaling activation that affect processes such as synaptic plasticity and insulin secretion.
11	ADCY9	adenylate cyclase 9	Adenylyl cyclase that catalyzes the formation of the signaling molecule cAMP in response to activation of G-protein-coupled receptors. Contributes to signaling cascades activated by CRH (corticotropin-releasing factor), corticosteroids and beta-adrenergic receptors.
12	ADCY10	adenylate cyclase 10	Catalyzes the formation of the signaling molecule cAMP. May function as sensor that mediates responses to changes in cellular bicarbonate and CO_2 levels.
13	PRKACA	protein kinase cAMP-activated catalytic subunit alpha	Phosphorylates HSF1; this phosphorylation promotes HSF1 nuclear localization and transcriptional activity upon heat shock.
14	PRKACB	protein kinase cAMP-activated catalytic subunit beta	Mediates cAMP-dependent signaling triggered by receptor binding to GPCRs. Protein kinase A (PKA) activation regulates diverse cellular processes such as cell proliferation, cell cycle, differentiation and regulation of microtubule dynamics, chromatin condensation and decondensation, nuclear envelope disassembly and reassembly, as well as regulation of intracellular transport mechanisms and ion flux. Regulates the abundance of compartmentalized pools of its regulatory subunits through phosphorylation of PJA2 which binds and ubiquitinates these subunits, leading to their subsequent proteolysis.
15	PRKACG	protein kinase cAMP-activated catalytic subunit gamma	Protein kinase A (PKA, aka cAMP-dependent protein kinase) is involved in the regulation of lipid and glucose metabolism and is a component of the signal transduction mechanism of certain GPCRs.
16	CREB1	cAMP responsive element binding protein 1	Involved in different cellular processes including the synchronization of circadian rhythmicity and the differentiation of adipose cells.
17	CREB3	cAMP responsive element binding protein 3	Translocates to the nucleus and activates unfolded protein response (UPR) target genes during endoplasmic reticulum (ER) stress response. Binds the cAMP response element (CRE) (consensus: 5'-GTGACGT[AG][AG]-3') and C/EBP sequences present in many promoters to activate transcription of the genes. Binds to the unfolded protein response element (UPRE) consensus sequences sites. Binds DNA to the 5'-CCAC[GA]-3'half of ERSE II (5'-ATTGG-N-CCACG-3').
18	CREB3L1	cAMP responsive element binding protein 3 like 1	In the absence of endoplasmic reticulum (ER) stress, inserted into ER membranes, with N-terminal DNA-binding and transcription activation domains oriented toward the cytosolic face of the membrane.
19	CREB3L2	cAMP responsive element binding protein 3 like 2	Transcription factor involved in unfolded protein response (UPR). In the absence of endoplasmic reticulum (ER) stress, inserted into ER membranes, with N-terminal DNA-binding and transcription activation domains oriented toward the cytosolic face of the membrane.
20	CREB3L3	cAMP responsive element binding protein 3 like 3	Transcription factor that may act during endoplasmic reticulum (ER) stress by activating unfolded protein response target genes. In acute inflammatory response, may activate expression of acute phase

			response (APR) genes. May be involved in growth suppression. Regulates FGF21 transcription
21	CREB3L4	cAMP responsive element binding protein 3 like 4	Transcriptional activator that may play a role in the unfolded protein response. Binds to the UPR element (UPRE) but not to CRE element.
22	CREB5	cAMP responsive element binding protein 5	Binds to the cAMP response element and activates transcription.
23	PRDM16	PR/SET domain 16	Binds DNA and functions as a transcriptional regulator.<br>Functions in the differentiation of brown adipose tissue (BAT) which is specialized in dissipating chemical energy in the form of heat in response to cold or excess feeding while white adipose tissue (WAT) is specialized in the storage of excess energy and the control of systemic metabolism.
24	ZNF516	zinc finger protein 516	Transcriptional regulator that binds to the promoter and activates the transcription of genes promoting brown adipose tissue (BAT) differentiation. Among brown adipose tissue-specific genes, binds the proximal region of the promoter of the UCP1 gene to activate its transcription and thereby regulate thermogenesis.
25	KDM1A	lysine demethylase 1A	Acts by oxidizing the substrate by FAD to generate the corresponding imine that is subsequently hydrolyzed.
26	UCP1	uncoupling protein 1	Mitochondrial protein responsible for thermogenic respiration, a specialized capacity of brown adipose tissue and beige fat that participates in non-shivering adaptive thermogenesis to temperature and diet variations and more generally to the regulation of energy balance.
27	KDM3A	lysine demethylase 3A	Plays a central role in histone code. Preferentially demethylates mono- and dimethylated H3 'Lys-9' residue, with a preference for dimethylated residue, while it has weak or no activity on trimethylated H3 'Lys-9'. Demethylation of Lys residue generates formaldehyde and succinate.
28	KDM3B	lysine demethylase 3B	Histone demethylase that specifically demethylates 'Lys-9' of histone H3, thereby playing a central role in histone code. Demethylation of Lys residue generates formaldehyde and succinate. May have tumor suppressor activity.
29	SMARCA2	SWI/SNF related, matrix associated, actin dependent regulator of chromatin, subfamily a, member 2	Involved in transcriptional activation and repression of select genes by chromatin remodeling (alteration of DNA-nucleosome topology).
30	SMARCA4	SWI/SNF related, matrix associated, actin dependent regulator of chromatin, subfamily a, member 4	Involved in transcriptional activation and repression of select genes by chromatin remodeling.<br>Component of the CREST-BRG1 complex, a multiprotein complex that regulates promoter activation by orchestrating the calcium-dependent release of a repressor complex and the recruitment of an activator complex.
31	SMARCB1	SWI/SNF related, matrix associated, actin dependent regulator of chromatin, subfamily b, member 1	Core component of the BAF (hSWI/SNF) complex.<br>Plays a key role in cell-cycle control and causes cell cycle arrest in G0/G1.

32	SMARCC1	SWI/SNF related, matrix associated, actin dependent regulator of chromatin subfamily c member 1	Involved in transcriptional activation and repression of select genes by chromatin remodeling.
33	SMARCC2	SWI/SNF related, matrix associated, actin dependent regulator of chromatin subfamily c member 2	Belongs to the neural progenitors-specific chromatin remodeling complex (npBAF complex) and the neuron-specific chromatin remodeling complex (nBAF complex). Critical regulator of myeloid differentiation, controlling granulocytopoiesis and the expression of genes involved in neutrophil granule formation
34	SMARCD1	SWI/SNF related, matrix associated, actin dependent regulator of chromatin, subfamily d, member 1	Has a strong influence on vitamin D-mediated transcriptional activity from an enhancer vitamin D receptor element.
35	SMARCD2	SWI/SNF related, matrix associated, actin dependent regulator of chromatin, subfamily d, member 2	Critical regulator of myeloid differentiation, controlling granulocytopoiesis and the expression of genes involved in neutrophil granule formation.
36	SMARCD3	SWI/SNF related, matrix associated, actin dependent regulator of chromatin, subfamily d, member 3	Involved in transcriptional activation and repression of select genes by chromatin remodeling.<br>Stimulates nuclear receptor mediated transcription.
37	SMARCE1	SWI/SNF related, matrix associated, actin dependent regulator of chromatin, subfamily e, member 1	Specifically interacts with the CoREST corepressor resulting in repression of neuronal specific gene promoters in non-neuronal cells.
38	ACTG1	actin gamma 1	Actins are highly conserved proteins that are involved in various types of cell motility and are ubiquitously expressed in all eukaryotic cells.
39	ACTB	actin beta	Actin exists in both monomeric (G-actin) and polymeric (F-actin) forms, both forms playing key functions, such as cell motility and contraction.
40	ACTL6A	actin like 6A	Putative core component of the chromatin remodeling INO80 complex which is involved in transcriptional regulation, DNA replication and probably DNA repair.
41	ACTL6B	actin like 6B	Essential for neuronal maturation and dendrite development
42	ARID1B	AT-rich interaction domain 1B	Involved in transcriptional activation and repression of select genes by chromatin remodeling.
43	ARID1A	AT-rich interaction domain 1A	Involved in transcriptional activation and repression of select genes by chromatin remodeling.<br>Plays a role regulating the activity of genes essential for dendrite growth.
44	DPF1	double PHD fingers 1	Important role in developing neurons by participating in regulation of cell survival, possibly as a neurospecific transcription factor.
45	DPF3	double PHD fingers 3	It acts as a tissue-specific anchor between histone acetylations and methylations and chromatin remodeling. It thereby probably plays an essential role in heart and skeletal muscle development.

46	PPARG	peroxisome proliferator activated receptor gamma	It therefore controls the peroxisomal beta-oxidation pathway of fatty acids. Key regulator of adipocyte differentiation and glucose homeostasis. ARF6 acts as a key regulator of the tissue-specific adipocyte P2 (aP2) enhancer.
47	MAP3K5	mitogen-activated protein kinase kinase kinase 5	Serine/threonine kinase which acts as an essential component of the MAP kinase signal transduction pathway. Plays an important role in the cascades of cellular responses evoked by changes in the environment. Mediates signal transduction of various stressors like oxidative stress as well as by receptor-48mediated inflammatory signals, such as the tumor necrosis fac49tor (TNF) or lipopolysaccharide (LPS).
48	MAP2K3	mitogen-activated protein kinase kinase 3	Part of a signaling cascade that begins with the activation of the adrenergic receptor ADRA1B and leads to the activation of MAPK14.
49	MAPK11	mitogen-activated protein kinase 11	MAPK11 is one of the four p38 MAPKs which play an important role in the cascades of cellular responses evoked by extracellular stimuli such as proinflammatory cytokines or physical stress leading to direct activation of transcription factors.
50	MAPK12	mitogen-activated protein kinase 12	MAPK12 is one of the four p38 MAPKs which play an important role in the cascades of cellular responses evoked by extracellular stimuli such as proinflammatory cytokines or physical stress leading to direct activation of transcription factors such as ELK1 and ATF2.
51	MAPK13	mitogen-activated protein kinase 13	MAPK13 is one of the four p38 MAPKs which play an important role in the cascades of cellular responses evoked by extracellular stimuli such as proinflammatory cytokines or physical stress leading to direct activation of transcription factors such as ELK1 and ATF2.<br>MAPK13 also phosphorylates and down-regulates PRKD1 during regulation of insulin secretion in pancreatic beta cells.
52	MAPK14	mitogen-activated protein kinase 14	Plays an essential role in developmental and stress-induced erythropoiesis, through regulation of EPO gene expression.<br>Isoform EXIP may play a role in the early onset of apoptosis.
53	PPARGC1A	PPARG coactivator 1 alpha	Greatly increases the transcriptional activity of PPARG and thyroid hormone receptor on the uncoupling protein promoter. Can regulate key mitochondrial genes that contribute to the program of adaptive thermogenesis.
54	ATF2	activating transcription factor 2	regulates the transcription of various genes, including those involved in anti-apoptosis, cell growth, and DNA damage response.
55	SIRT6	sirtuin 6	Required for normal IGF1 serum levels and normal glucose homeostasis
56	FGF21	fibroblast growth factor 21	Stimulates glucose uptake in differentiated adipocytes via the induction of glucose transporter SLC2A1/GLUT1 expression (but not SLC2A4/GLUT4 expression). Activity requires the presence of KLB.
57	PLIN1	perilipin 1	Modulator of adipocyte lipid metabolism. Coats lipid storage droplets to protect them from breakdown by hormone-sensitive lipase (HSL). May modulate lipolysis and triglyceride levels.
58	LIPE	lipase E, hormone sensitive type	Lipase with broad substrate specificity, catalyzing the hydrolysis of triacylglycerols (TAGs), diacylglycerols (DAGs), monoacylglycerols (MAGs), cholesteryl esters and retinyl esters
59	MGLL	monoglyceride lipase	Converts monoacylglycerides to free fatty acids and glycerol. Regulates the levels of fatty acids that serve as signaling molecules and promote cancer cell migration, invasion and tumor growth.

60	PRKAA1	protein kinase AMP-activated catalytic subunit alpha 1	Inhibits protein, carbohydrate and lipid biosynthesis, as well as cell growth and proliferation.
61	PRKAA2	protein kinase AMP-activated catalytic subunit alpha 2	Can inhibit the non-autophagy complex by phosphorylating PIK3C3 and can activate the pro-autophagy complex by phosphorylating BECN1.
62	PRKAB1	protein kinase AMP-activated non-catalytic subunit beta 1	Plays a key role in regulating cellular energy metabolism.<br>Inhibits protein, carbohydrate and lipid biosynthesis, as well as cell growth and proliferation.
63	PRKAB2	protein kinase AMP-activated non-catalytic subunit beta 2	AMPK acts via direct phosphorylation of metabolic enzymes, and by longer-term effects via phosphorylation of transcription regulators.
64	PRKAG1	protein kinase AMP-activated non-catalytic subunit gamma 1	Inhibits protein, carbohydrate and lipid biosynthesis, as well as cell growth and proliferation.
65	PRKAG3	protein kinase AMP-activated non-catalytic subunit gamma 3	An energy sensor protein kinase that plays a key role in regulating cellular energy metabolism.
66	PRKAG2	protein kinase AMP-activated non-catalytic subunit gamma 2	Acts as a regulator of cellular polarity by remodeling the actin cytoskeleton; probably by indirectly activating myosin.
67	GCG	glucagon	Plays a key role in glucose metabolism and homeostasis. Regulates blood glucose by increasing gluconeogenesis and decreasing glycolysis. A counterregulatory hormone of insulin, raises plasma glucose levels in response to insulin-induced hypoglycemia. Plays an important role in initiating and maintaining hyperglycemic conditions in diabetes.
68	BMP8B	bone morphogenetic protein 8b	Induces cartilage and bone formation. May be the osteoinductive factor responsible for the phenomenon of epithelial osteogenesis.
69	BMP8A	bone morphogenetic protein 8b	Signaling protein involved in regulation of thermogenesis and energy balance.
70	CNR1	cannabinoid receptor 1	Induces leptin production in adipocytes and reduces LRP2-mediated leptin clearance in the kidney, hence participating in hyperleptinemia.
71	NPPA	natriuretic peptide A	Role in cardio-renal homeostasis through regulation of natriuresis, diuresis, and vasodilation.
72	NPPB	natriuretic peptide B	Cardiac hormone that plays a key role in mediating cardio-renal homeostasis.
73	NPR1	natriuretic peptide receptor 1	May act as a substrate-specific adapter of an E3 ubiquitin-protein ligase complex (CUL3-RBX1-BTB) which mediates the ubiquitination and subsequent proteasomal degradation of target proteins.
74	PRKG1	protein kinase cGMP-dependent 1	Serine/threonine protein kinase that acts as key mediator of the nitric oxide (NO)/cGMP signaling pathway.
75	PRKG2	protein kinase cGMP-dependent 2	Crucial regulator of intestinal secretion and bone growth.
76	KLB	klotho beta	Contributes to the transcriptional repression of cholesterol 7-alpha-hydroxylase (CYP7A1), the rate-limiting enzyme in bile acid synthesis. Probably inactive as a glycosidase. Increases the ability of FGFR1 and FGFR4 to bind FGF21.
77	FGFR1	fibroblast growth factor receptor 1	Tyrosine-protein kinase that acts as cell-surface receptor for fibroblast growth factors and plays an essential role in the regulation of embryonic development, cell proliferation, differentiation and migration.

78	FRS2	fibroblast growth factor receptor substrate 2	Lays an important role in the activation of MAP kinases and in the phosphorylation of PIK3R1.
79	GRB2	growth factor receptor bound protein 2	Adapter protein that provides a critical link between cell surface growth factor receptors and the Ras signaling pathway.
80	SOS1	SOS Ras/Rac guanine nucleotide exchange factor 1	Promotes the exchange of Ras-bound GDP by GTP.<br>Catalytic component of a trimeric complex.
81	SOS2	SOS Ras/Rho guanine nucleotide exchange factor 2	Promotes the exchange of Ras-bound GDP by GTP.
82	HRAS	HRas proto-oncogene, GTPase	Involved in the activation of Ras protein signal transduction.
83	KRAS	KRAS proto-oncogene, GTPase	Plays an important role in the regulation of cell proliferation.<br>Plays a role in promoting oncogenic events by inducing transcriptional silencing of tumor suppressor genes (TSGs) in colorectal cancer (CRC) cells in a ZNF304-dependent manner.
84	NRAS	NRAS proto-oncogene, GTPase	Ras proteins bind GDP/GTP and possess intrinsic GTPase activity.
85	RPS6KA3	ribosomal protein S6 kinase A3	Mediates cellular proliferation, survival, and differentiation by modulating mTOR signaling and repressing pro-apoptotic function of BAD and DAPK1.
86	RPS6KA1	ribosomal protein S6 kinase A1	Is involved in cell cycle regulation by phosphorylating the CDK inhibitor CDKN1B.
87	RPS6KA2	ribosomal protein S6 kinase A2	Serine/threonine-protein kinase that acts downstream of ERK (MAPK1/ERK2 and MAPK3/ERK1) signaling and mediates mitogenic and stress-induced activation of transcription factors, regulates translation, and mediates cellular proliferation, survival, and differentiation. May function as tumor suppressor in epithelial ovarian cancer cells.
88	RPS6KA6	ribosomal protein S6 kinase A6	Constitutively active serine/threonine-protein kinase that exhibits growth-factor-independent kinase activity and that may participate in p53/TP53-dependent cell growth arrest signaling and play an inhibitory role during embryogenesis.
89	TSC1	TSC complex subunit 1	In complex with TSC2, inhibits the nutrient-mediated or growth factor-stimulated phosphorylation of S6K1 and EIF4EBP1 by negatively regulating mTORC1 signaling.
90	TSC2	TSC complex subunit 2	Acts as a GTPase-activating protein (GAP) for the small GTPase RHEB, a direct activator of the protein kinase activity of mTORC1.
91	RHEB	Ras homolog, mTORC1 binding	Activates the protein kinase activity of mTORC1, and thereby plays a role in the regulation of apoptosis. Stimulates the phosphorylation of S6K1 and EIF4EBP1 through activation of mTORC1 signaling. Has low intrinsic GTPase activity.
92	RPTOR	regulatory associated protein of MTOR complex 1	Involved in the control of the mammalian target of rapamycin complex 1 (mTORC1) activity which regulates cell growth and survival, and autophagy in response to nutrient and hormonal signals; functions as a scaffold for recruiting mTORC1 substrates.
93	MTOR	mechanistic target of rapamycin kinase	Central regulator of cellular metabolism, growth and survival in response to hormones, growth factors, nutrients, energy and stress signals
94	MLST8	MTOR associated protein, LST8 homolog	Subunit of both mTORC1 and mTORC2, which regulates cell growth and survival in response to nutrient and hormonal signals.

95	AKT1S1	AKT1 substrate 1	Subunit of mTORC1, which regulates cell growth and survival in response to nutrient and hormonal signals.
96	RPS6KB1	ribosomal protein S6 kinase B1	Regulates protein synthesis through phosphorylation of EIF4B, RPS6 and EEF2K, and contributes to cell survival by repressing the pro-apoptotic function of BAD.
97	RPS6KB2	ribosomal protein S6 kinase B2	Phosphorylates specifically ribosomal protein S6.
98	RPS6	ribosomal protein S6	Component of the 40S small ribosomal subunit.<br>Plays an important role in controlling cell growth and proliferation through the selective translation of particular classes of mRNA.
99	ACSL6	acyl-CoA synthetase long chain family member 6	Catalyzes the conversion of long-chain fatty acids to their active form acyl-CoA for both synthesis of cellular lipids, and degradation via beta-oxidation.<br>Plays an important role in fatty acid metabolism in brain and the acyl-CoAs produced may be utilized exclusively for the synthesis of the brain lipid.
100	ACSL4	acyl-CoA synthetase long chain family member 4	Catalyzes the conversion of long-chain fatty acids to their active form acyl-CoA for both synthesis of cellular lipids, and degradation via beta-oxidation.
101	ACSL1	acyl-CoA synthetase long chain family member 1	Catalyzes the conversion of long-chain fatty acids to their active form acyl-CoAs for both synthesis of cellular lipids, and degradation via beta-oxidation
102	ACSL5	acyl-CoA synthetase long chain family member 5	ACSL5 may activate fatty acids from exogenous sources for the synthesis of triacylglycerol destined for intracellular storage.
103	ACSL3	acyl-CoA synthetase long chain family member 3	Required for the incorporation of fatty acids into phosphatidylcholine, the major phospholipid located on the surface of VLDL.<br>Has mainly an anabolic role in energy metabolism. Mediates hepatic lipogenesis.
104	ND1	NADH dehydrogenase subunit 1	Functions in the transfer of electrons from NADH to the respiratory chain.
105	ND2	NADH dehydrogenase subunit 2	Core subunit of the mitochondrial membrane respiratory chain NADH dehydrogenase (Complex I) that is believed to belong to the minimal assembly required for catalysis.
106	ND3	NADH dehydrogenase subunit 3	Functions in the transfer of electrons from NADH to the respiratory chain.
107	ND4	NADH dehydrogenase subunit 4	Core subunit of the mitochondrial membrane respiratory chain NADH dehydrogenase (Complex I) that is believed to belong to the minimal assembly required for catalysis.
108	ND4L	NADH dehydrogenase subunit 4L	Core subunit of the mitochondrial membrane respiratory chain NADH dehydrogenase (Complex I) that is believed to belong to the minimal assembly required for catalysis. Complex I functions in the transfer of electrons from NADH to the respiratory chain.
109	ND5	NADH dehydrogenase subunit 5	Core subunit of the mitochondrial membrane respiratory chain NADH dehydrogenase (Complex I) that is believed to belong to the minimal assembly required for catalysis.
110	ND6	NADH dehydrogenase subunit 6	Core subunit of the mitochondrial membrane respiratory chain NADH dehydrogenase (Complex I) that is believed to belong to the minimal assembly required for catalysis.
111	NDUFS1	NADH: ubiquinone oxidoreductase core subunit S1	Essential for catalyzing the entry and efficient transfer of electrons within complex I.

112	NDUFS2	NADH: ubiquinone oxidoreductase core subunit S2	Redox-sensitive, critical component of the oxygen-sensing pathway in the pulmonary vasculature which plays a key role in acute pulmonary oxygen-sensing and hypoxic pulmonary vasoconstriction.<br>Plays an important role in carotid body sensing of hypoxia
113	NDUFS3	NADH: ubiquinone oxidoreductase core subunit S3	Core subunit of the mitochondrial membrane respiratory chain NADH dehydrogenase (Complex I).
114	NDUFS4	NADH: ubiquinone oxidoreductase subunit S4	Accessory subunit of the mitochondrial membrane respiratory chain NADH dehydrogenase (Complex I), that is believed not to be involved in catalysis.
115	NDUFS5	NADH: ubiquinone oxidoreductase subunit S5	Accessory subunit of the mitochondrial membrane respiratory chain NADH dehydrogenase (Complex I), that is believed not to be involved in catalysis.
116	NDUFS6	NADH: ubiquinone oxidoreductase subunit S6	Accessory subunit of the mitochondrial membrane respiratory chain NADH dehydrogenase (Complex I), that is believed not to be involved in catalysis.
117	NDUFS7	NADH: ubiquinone oxidoreductase core subunit S7	Core subunit of the mitochondrial membrane respiratory chain NADH dehydrogenase (Complex I) which catalyzes electron transfer from NADH through the respiratory chain, using ubiquinone as an electron acceptor.
118	NDUFS8	NADH: ubiquinone oxidoreductase core subunit S8	Core subunit of the mitochondrial membrane respiratory chain NADH dehydrogenase (Complex I) which catalyzes electron transfer from NADH through the respiratory chain, using ubiquinone as an electron acceptor.<br>Essential for the catalytic activity and assembly of complex I.
119	NDUFV1	NADH: ubiquinone oxidoreductase core subunit V1	Complex I functions in the transfer of electrons from NADH to the respiratory chain.
120	NDUFV2	NADH: ubiquinone oxidoreductase core subunit V2	Complex I functions in the transfer of electrons from NADH to the respiratory chain.
121	NDUFV3	NADH: ubiquinone oxidoreductase subunit V3	May be the terminally assembled subunit of Complex I.
122	NDUFA1	NADH: ubiquinone oxidoreductase subunit A1	Accessory subunit of the mitochondrial membrane respiratory chain NADH dehydrogenase (Complex I).
123	NDUFA2	NADH: ubiquinone oxidoreductase subunit A2	Complex I functions in the transfer of electrons from NADH to the respiratory chain.
124	NDUFA3	NADH: ubiquinone oxidoreductase subunit A3	Complex I functions in the transfer of electrons from NADH to the respiratory chain.
125	NDUFA4	NDUFA4 mitochondrial complex associated	Component of the cytochrome c oxidase, the last enzyme in the mitochondrial electron transport chain which drives oxidative phosphorylation.
126	NDUFA4L2	NDUFA4 mitochondrial complex associated like 2	Electron transport chain<br>Proton transmembrane transport
127	NDUFA5	NADH: ubiquinone oxidoreductase subunit A5	Accessory subunit of the mitochondrial membrane respiratory chain NADH dehydrogenase (Complex I), that is believed not to be involved in catalysis.

128	NDUFA6	NADH: ubiquinone oxidoreductase subunit A6	Accessory subunit of the mitochondrial membrane respiratory chain NADH dehydrogenase (Complex I), that is believed not to be involved in catalysis.
129	NDUFA7	NADH: ubiquinone oxidoreductase subunit A7	Accessory subunit of the mitochondrial membrane respiratory chain NADH dehydrogenase (Complex I), that is believed not to be involved in catalysis.
130	NDUFA8	NADH: ubiquinone oxidoreductase subunit A8	The human NDUFA8 gene codes for a subunit of Complex I of the respiratory chain, which transfers electrons from NADH to ubiquinone.
131	NDUFA9	NADH: ubiquinone oxidoreductase subunit A9	The human NDUFA9 gene codes for a subunit of Complex I of the respiratory chain, which transfers electrons from NADH to ubiquinone.
132	NDUFA10	NADH: ubiquinone oxidoreductase subunit A10	The human NDUFA10 gene codes for a subunit of Complex I of the respiratory chain, which transfers electrons from NADH to ubiquinone.
133	NDUFAB1	NADH: ubiquinone oxidoreductase subunit AB1	Carrier of the growing fatty acid chain in fatty acid biosynthesis.
134	NDUFA11	NADH: ubiquinone oxidoreductase subunit A11	The human NDUFA11 gene codes for a subunit of Complex I of the respiratory chain, which transfers electrons from NADH to ubiquinone.
135	NDUFA12	NADH: ubiquinone oxidoreductase subunit A12	The human NDUFA12 gene codes for a subunit of Complex I of the respiratory chain, which transfers electrons from NADH to ubiquinone.
136	NDUFA13	NADH: ubiquinone oxidoreductase subunit A13	Prevents the transactivation of STAT3 target genes. May play a role in CARD15-mediated innate mucosal responses and serve to regulate intestinal epithelial cell responses to microbes.
137	NDUFB1	NADH: ubiquinone oxidoreductase subunit B1	Accessory subunit of the mitochondrial membrane respiratory chain NADH dehydrogenase (Complex I) that is believed not to be involved in catalysis.
138	NDUFB2	NADH: ubiquinone oxidoreductase subunit B2	NDUFB2 is an accessory subunit of the complex that is believed not to be involved in catalysis.
139	NDUFB3	NADH: ubiquinone oxidoreductase subunit B3	The human NDUFB3 gene codes for a subunit of Complex I of the respiratory chain, which transfers electrons from NADH to ubiquinone.
140	NDUFB4	NADH: ubiquinone oxidoreductase subunit B4	NDUFB4 is an accessory subunit of the complex that is believed not to be involved in catalysis.
141	NDUFB5	NADH: ubiquinone oxidoreductase subunit B5	Accessory subunit of the mitochondrial membrane respiratory chain NADH dehydrogenase (Complex I), that is believed not to be involved in catalysis.
142	NDUFB6	NADH: ubiquinone oxidoreductase subunit B6	The protein encoded by this gene is an accessory subunit of the multi subunit NADH: ubiquinone oxidoreductase (complex I) that is not directly involved in catalysis.
143	NDUFB7	NADH: ubiquinone oxidoreductase subunit B7	The protein encoded by this gene is an accessory subunit of the multi subunit NADH: ubiquinone oxidoreductase (complex I) that is not directly involved in catalysis.
144	NDUFB8	NADH: ubiquinone oxidoreductase subunit B8	Accessory subunit of the mitochondrial membrane respiratory chain NADH dehydrogenase (Complex I), that is believed not to be involved in catalysis. Complex I functions in the transfer of electrons from NADH to the respiratory chain.

145	NDUFB9	NADH: ubiquinone oxidoreductase subunit B9	The protein encoded by this gene is an accessory subunit of the multi subunit NADH: ubiquinone oxidoreductase (complex I) that is not directly involved in catalysis.
146	NDUFB10	NADH: ubiquinone oxidoreductase subunit B10	The protein encoded by this gene is an accessory subunit of the multi subunit NADH: ubiquinone oxidoreductase (complex I) that is not directly involved in catalysis.
147	NDUFB11	NADH: ubiquinone oxidoreductase subunit B11	Accessory subunit of the mitochondrial membrane respiratory chain NADH dehydrogenase (Complex I), that is believed not to be involved in catalysis. Complex I functions in the transfer of electrons from NADH to the respiratory chain.
148	NDUFC1	NADH: ubiquinone oxidoreductase subunit C1	NADH dehydrogenase [ubiquinone] 1 subunit C1, mitochondrial is an enzyme that in humans is encoded by the NDUFC1 gene.
149	NDUFC2	NADH: ubiquinone oxidoreductase subunit C2	The NDUF2 gene encodes one of the subunits of complex I, the first and largest complex of the mitochondrial respiratory chain.
150	NDUFC2-KCTD14	NDUFC2-KCTD14 readthrough	Accessory subunit of the mitochondrial membrane respiratory chain NADH dehydrogenase (Complex I), that is believed not to be involved in catalysis. Complex I functions in the transfer of electrons from NADH to the respiratory chain.
151	NDUFAF1	NADH: ubiquinone oxidoreductase complex assembly factor 1	Chaperone protein involved in early stages of the assembly of mitochondrial NADH: ubiquinone oxidoreductase complex (complex I)
152	NDUFAF2	NADH: ubiquinone oxidoreductase complex assembly factor 2	Acts as a molecular chaperone for mitochondrial complex I assembly.
153	NDUFAF3	NADH: ubiquinone oxidoreductase complex assembly factor 3	Essential factor for the assembly of mitochondrial NADH: ubiquinone oxidoreductase complex (complex I).
154	NDUFAF4	NADH: ubiquinone oxidoreductase complex assembly factor 4	Involved in the assembly of mitochondrial NADH: ubiquinone oxidoreductase complex (complex I).<br>May be involved in cell proliferation and survival of hormone-dependent tumor cells. May be a regulator of breast tumor cell invasion.
155	NDUFAF5	NADH: ubiquinone oxidoreductase complex assembly factor 5	Arginine hydroxylase involved in the assembly of mitochondrial NADH: ubiquinone oxidoreductase complex (complex I, MT-ND1) at early stages.
156	NDUFAF6	NADH: ubiquinone oxidoreductase complex assembly factor 6	Involved in the assembly of mitochondrial NADH: ubiquinone oxidoreductase complex (complex I) at early stages. May play a role in the biogenesis of MT-ND1.
157	NDUFAF7	NADH: ubiquinone oxidoreductase complex assembly factor 7	Arginine methyltransferase involved in the assembly or stability of mitochondrial NADH: ubiquinone oxidoreductase complex (complex I).
158	NDUFAF8	NADH: ubiquinone oxidoreductase complex assembly factor 8	Involved in the assembly of mitochondrial NADH: ubiquinone oxidoreductase complex (complex I, MT-ND1).<br>Required to stabilize NDUFAF5.
159	SDHA	succinate dehydrogenase complex flavoprotein subunit A	Flavoprotein (FP) subunit of succinate dehydrogenase (SDH) that is involved in complex II of the mitochondrial electron transport chain and is responsible for transferring electrons from succinate to ubiquinone (coenzyme Q).<br>Can act as a tumor suppressor.

160	SDHB	succinate dehydrogenase complex iron sulfur subunit B	Iron-sulfur protein (IP) subunit of succinate dehydrogenase (SDH) that is involved in complex II of the mitochondrial electron transport chain and is responsible for transferring electrons from succinate to ubiquinone (coenzyme Q).
161	SDHC	succinate dehydrogenase complex subunit C	Membrane-anchoring subunit of succinate dehydrogenase (SDH) that is involved in complex II of the mitochondrial electron transport chain and is responsible for transferring electrons from succinate to ubiquinone (coenzyme Q).
162	SDHD	succinate dehydrogenase complex subunit D	SDHD forms part of the transmembrane protein dimer with SDHC that anchors Complex II to the inner mitochondrial membrane.
163	UQCRFS1	ubiquinol-cytochrome c reductase, Rieske iron-sulfur polypeptide 1	Component of the ubiquinol-cytochrome c oxidoreductase, a multi subunit transmembrane complex that is part of the mitochondrial electron transport chain which drives oxidative phosphorylation. The Rieske protein is a high potential 2Fe-2S protein.
164	CYTB	cytochrome b	Component of the ubiquinol-cytochrome c reductase complex (complex III or cytochrome b-c1 complex) that is part of the mitochondrial respiratory chain. The b-c1 complex mediates electron transfer from ubiquinol to cytochrome c. Contributes to the generation of a proton gradient across the mitochondrial membrane that is then used for ATP synthesis.
165	CYC1	cytochrome c1	CYC1 encodes a protein that is located in the inner mitochondrial membrane and is part of Ubiquinol Cytochrome c Reductase (complex III). The encoded protein, CYC1, is a respiratory subunit of the cytochrome bc1 complex, which plays an important role in the mitochondrial respiratory chain by transferring electrons from the Rieske iron-sulfur protein to cytochrome c.
166	UQCRC1	ubiquinol-cytochrome c reductase core protein 1	Component of the ubiquinol-cytochrome c oxidoreductase, a multi subunit transmembrane complex that is part of the mitochondrial electron transport chain which drives oxidative phosphorylation.
167	UQCRC2	ubiquinol-cytochrome c reductase core protein 2	Component of the ubiquinol-cytochrome c oxidoreductase, a multi subunit transmembrane complex that is part of the mitochondrial electron transport chain which drives oxidative phosphorylation.
168	UQCRH	ubiquinol-cytochrome c reductase hinge protein	Its gene product is a subunit of the respiratory chain protein Ubiquinol Cytochrome c Reductase.
169	UQCRHL	ubiquinol-cytochrome c reductase hinge protein like	May be a component of the ubiquinol-cytochrome c reductase complex (complex III or cytochrome b-c1 complex), which is part of the mitochondrial respiratory chain. This protein may mediate formation of the complex between cytochromes c and c1.
170	UQCRB	ubiquinol-cytochrome c reductase binding protein	Component of the ubiquinol-cytochrome c oxidoreductase, a multi subunit transmembrane complex that is part of the mitochondrial electron transport chain which drives oxidative phosphorylation.
171	UQCRQ	ubiquinol-cytochrome c reductase complex III subunit VII	This gene encodes a ubiquinone-binding protein of low molecular mass. It is a small core-associated protein and a subunit of ubiquinol-cytochrome c reductase complex III, which is part of the mitochondrial respiratory chain.
172	UQCR10	ubiquinol-cytochrome c reductase, complex III subunit X	Its gene product is a subunit of the respiratory chain protein Ubiquinol Cytochrome c Reductase (UQCR, Complex III or Cytochrome bc1 complex), which consists of the products of one mitochondrially encoded gene, MTCYTB (mitochondrial cytochrome b) and ten nuclear genes

173	UQCR11	ubiquinol-cytochrome c reductase, complex III subunit XI	The UQCR11 protein may function as a binding factor for the iron-sulfur protein in Complex III, which is ubiquitous in human cells.
174	COX1	cytochrome c oxidase subunit I	Component of the cytochrome c oxidase, the last enzyme in the mitochondrial electron transport chain which drives oxidative phosphorylation.
175	COX2	cytochrome c oxidase subunit II	PTGS2 (COX-2), converts arachidonic acid (AA) to prostaglandin endoperoxide H2. PGHSs are targets for NSAIDs and PTGS2 (COX-2) specific inhibitors called coxibs.
176	COX3	cytochrome c oxidase subunit III	Component of the cytochrome c oxidase, the last enzyme in the mitochondrial electron transport chain which drives oxidative phosphorylation.
177	COX4I2	cytochrome c oxidase subunit 4I2	Subunit IV is the largest nuclear encoded subunit which plays a pivotal role in COX regulation.<br>Role for COX4I2 in the optimization of the electron transfer chain under different conditions.
178	COX4I1	cytochrome c oxidase subunit 4I1	Cytochrome c oxidase is the component of the respiratory chain that catalyzes the reduction of oxygen to water.
179	COX5A	cytochrome c oxidase subunit 5A	COX5A (this gene) and COX5B are involved in the regulation of cancer cell metabolism by Bcl-2. COX5A interacts specifically with Bcl-2, but not with other members of the Bcl-2 family, such as Bcl-xL, Bax or Bak.
180	COX5B	cytochrome c oxidase subunit 5B	COX5A (this gene) and COX5B are involved in the regulation of cancer cell metabolism by Bcl-2. COX5A interacts specifically with Bcl-2, but not with other members of the Bcl-2 family, such as Bcl-xL, Bax or Bak.
181	COX6A1	cytochrome c oxidase subunit 6A1	A mutation leading to a 5 base pair deletion in the COX6A1 gene is associated with Charcot-Marie-Tooth disease.
182	COX6A2	cytochrome c oxidase subunit 6A2	The Trans-activator of transcription protein (Tat) of human immunodeficiency virus (HIV) inhibits cytochrome c oxidase (COX) activity in permeabilized mitochondria isolated from both mouse and human liver, heart, and brain samples.
183	COX6B1	cytochrome c oxidase subunit 6B1	Mutations affecting the COX6B1 gene are associated with mitochondrial complex IV deficiency (MT-C4D), a disorder of the mitochondrial respiratory chain with heterogeneous clinical manifestations, ranging from isolated myopathy to severe multisystem disease affecting several tissues and organs.
184	COX6B2	cytochrome c oxidase subunit 6B2	Cytochrome c oxidase (COX) is the terminal enzyme of the mitochondrial respiratory chain.
185	COX6C	cytochrome c oxidase subunit 6C	This nuclear gene encodes subunit VIc, which has 77% amino acid sequence identity with mouse COX subunit VIc. This gene is up-regulated in prostate cancer cells. A pseudogene COX6CP1 has been found on chromosomes 16p12
186	COX7A1	cytochrome c oxidase subunit 7A1	This nuclear gene encodes polypeptide 1 (muscle isoform) of subunit VIIa and the polypeptide 1 is present only in muscle tissues. Other polypeptides of subunit VIIa are present in both muscle and nonmuscle tissues, and are encoded by different genes.
187	COX7A2	cytochrome c oxidase subunit 7A2	This nuclear gene encodes polypeptide 2 (liver isoform) of subunit VIIa and the polypeptide 2 is present in both muscle and non-muscle tissues.
188	COX7A2L	cytochrome c oxidase subunit 7A2 like	This nuclear gene encodes a protein similar to polypeptides 1 and 2 of subunit VIIa in the C-terminal region, and also highly similar to the mouse Sig81 protein sequence.

189	COX7B	cytochrome c oxidase subunit 7B	The COX7B nuclear gene encodes subunit 7B, which is located on the inner mitochondrial membrane in association with several other proteins encompassing the COX complex.
190	COX7B2	cytochrome c oxidase subunit 7B2	This protein is involved in the pathway oxidative phosphorylation, which is part of energy metabolism.
191	COX7C	cytochrome c oxidase subunit 7C	This nuclear gene encodes subunit VIIc, which shares 87% and 85% amino acid sequence identity with mouse and bovine COX VIIc, respectively, and is found in all tissues.
192	COX8C	cytochrome c oxidase subunit 8C	This protein is involved in the pathway oxidative phosphorylation, which is part of energy metabolism.
193	COX8A	cytochrome c oxidase subunit 8A	COX8A is a subunit of cytochrome c oxidase and its function is important for the efficacy of complex IV. Mutations in COX8A can affect complex IV of the electron transport chain, resulting in complex IV deficiency.
194	COX10	cytochrome c oxidase assembly factor heme A:farnesyltransferase COX10	The protein encoded by COX10 is an assembly factor essential to COX synthesis, participating in the first step of the mitochondrial heme A biosynthetic pathway. It catalyzes the farnesylation of the vinyl group position C2 of protoheme (heme B) and converts it to heme O.
195	COX11	cytochrome c oxidase copper chaperone COX11	Exerts its effect at some terminal stage of cytochrome c oxidase synthesis, probably by being involved in the insertion of the copper B into subunit I.
196	COX14	cytochrome c oxidase assembly factor COX14	The COX14 gene encodes for a core protein component of the MITRAC (mitochondrial translation regulation assembly intermediate of cytochrome c oxidase complex) complex, which is required for the proper regulation of complex IV assembly.
197	COX15	cytochrome c oxidase assembly homolog COX15	COX15 is one of the cytochrome c oxidase (COX) assembly factors identified in yeast, playing a key role in the biosynthetic pathway of mitochondrial heme A, the prosthetic group of cytochrome a and a3. COX15 in yeast mediates hydroxylation of the methyl group at the C-8 position of the heme O molecule to form heme A.
198	COX16	cytochrome c oxidase assembly factor COX16	Required for the assembly of the mitochondrial respiratory chain complex IV (CIV), also known as cytochrome c oxidase.
199	COX17	cytochrome c oxidase copper chaperone COX1	Cytochrome c oxidase copper chaperone is a protein that in humans is encoded by the COX17 gene.<br>This nuclear gene encodes a protein which is not a structural subunit, but may be involved in the recruitment of copper to mitochondria for incorporation into the COX apoenzyme.
200	COX18	cytochrome c oxidase assembly factor COX18	Plays a role in MT-CO2/COX2 maturation following the COX20-mediated stabilization of newly synthesized MT-CO2/COX2 protein and before the action of the metallochaperones SCO1/2. Essential for the assembly and stability of the mitochondrial respiratory chain complex IV.
201	COX19	cytochrome c oxidase assembly factor COX19	Protein required for cytochrome c oxidase assembly; located in the cytosol and mitochondrial intermembrane space; putative copper metallochaperone that delivers copper to cytochrome c oxidase; contains twin cysteine-x9-cysteine motifs.
202	COX20	cytochrome c oxidase assembly factor COX20	Essential for the assembly of the mitochondrial respiratory chain complex IV (CIV), also known as cytochrome c oxidase.
203	COA1	cytochrome c oxidase assembly factor 1 homolog	Component of the MITRAC (mitochondrial translation regulation assembly intermediate of cytochrome c oxidase complex) complex, that regulates cytochrome c oxidase assembly.

204	COA3	cytochrome c oxidase assembly factor 3	Core component of the MITRAC (mitochondrial translation regulation assembly intermediate of cytochrome c oxidase complex) complex, that regulates cytochrome c oxidase assembly.
205	COA4	cytochrome c oxidase assembly factor 4 homolog	Involved in cytochrome c oxidase assembly or stability.
206	COA5	cytochrome c oxidase assembly factor 5	Involved in an early step of the mitochondrial complex IV assembly process.
207	COA6	cytochrome c oxidase assembly factor 6	Involved in the maturation of the mitochondrial respiratory chain complex IV subunit MT-CO2/COX2. Thereby, may regulate early steps of complex IV assembly.
208	COA7	cytochrome c oxidase assembly factor 7	Required for assembly of mitochondrial respiratory chain complex I and complex IV.
209	ATP5F1A	ATP synthase F1 subunit alpha	Mitochondrial membrane ATP synthase (F_1F_0 ATP synthase or Complex V) produces ATP from ADP in the presence of a proton gradient across the membrane which is generated by electron transport complexes of the respiratory chain.
210	ATP5F1B	ATP synthase F1 subunit beta	This gene encodes a subunit of mitochondrial ATP synthase.<br>The catalytic portion of mitochondrial ATP synthase consists of 5 different subunits (alpha, beta, gamma, delta, and epsilon) assembled with a stoichiometry of 3 alpha, 3 beta, and a single representative of the other 3.
211	ATP5F1C	ATP synthase F1 subunit gamma	This gene encodes the gamma subunit of the catalytic core.
212	ATP5F1D	ATP synthase F1 subunit delta	This gene encodes a subunit of the mitochondrial ATP synthase (Complex V) of the mitochondrial respiratory chain, which is necessary for the catalysis of ATP synthesis.
213	ATP5F1E	ATP synthase F1 subunit epsilon	This gene encodes the epsilon subunit of the catalytic core.
214	ATP5PO	ATP synthase peripheral stalk subunit OSCP	Mitochondrial membrane ATP synthase (F_1F_0 ATP synthase or Complex V) produces ATP from ADP in the presence of a proton gradient across the membrane which is generated by electron transport complexes of the respiratory chain.
215	ATP6	ATP synthase F0 subunit 6	This subunit is a key component of the proton channel, and may play a direct role in the translocation of protons across the membrane. Catalysis in the F_1 complex depends upon the rotation of the central stalk and F_0c-ring, which in turn is driven by the flux of protons through the membrane via the interface between the F0 c-ring and subunit A.
216	ATP5PB	ATP synthase peripheral stalk-membrane subunit b	The b subunits are part of the peripheral stalk that links the F1 and FO complexes together, and which acts as a stator to prevent certain subunits from rotating with the central rotary element.
217	ATP5MC1	ATP synthase membrane subunit c locus 1	This gene encodes a subunit of mitochondrial ATP synthase. Mitochondrial ATP synthase catalyzes ATP synthesis, utilizing an electrochemical gradient of protons across the inner membrane during oxidative phosphorylation.
218	ATP5MC2	ATP synthase membrane subunit c locus 2	This gene encodes a subunit of mitochondrial ATP synthase. Mitochondrial ATP synthase catalyzes ATP synthesis, utilizing an electrochemical gradient of protons across the inner membrane during oxidative phosphorylation.
219	ATP5MC3	ATP synthase membrane subunit c locus 3	The ATP5MC3 gene is one of three human paralogs that encode membrane subunit c of the mitochondrial ATP synthase.

220	ATP5PD	ATP synthase peripheral stalk subunit d	Mitochondrial ATP synthase catalyzes ATP synthesis, utilizing an electrochemical gradient of protons across the inner membrane during oxidative phosphorylation.
221	ATP5ME	ATP synthase membrane subunit e	The F1 complex consists of 5 different subunits (alpha, beta, gamma, delta, and epsilon) assembled in a ratio of 3 alpha, 3 beta, and a single representative of the other 3.
222	ATP5MF	ATP synthase membrane subunit f	Mitochondrial ATP synthase catalyzes ATP synthesis, utilizing an electrochemical gradient of protons across the inner membrane during oxidative phosphorylation.
223	ATP5PF	ATP synthase peripheral stalk subunit F6	The F6 subunit is part of the peripheral stalk that links the F_1 and F_O complexes together, and which acts as a stator to prevent certain subunits from rotating with the central rotary element.
224	ATP8	ATP synthase F0 subunit 8	The MT-ATP8 gene encodes a subunit of mitochondrial ATP synthase, located within the thylakoid membrane and the inner mitochondrial membrane. Mitochondrial ATP synthase catalyzes ATP synthesis, utilizing an electrochemical gradient of protons across the inner membrane during oxidative phosphorylation.
225	ATP5MG	ATP synthase membrane subunit g	This gene encodes the g subunit of the F0 complex.
226	CPT2	carnitine palmitoyltransferase 2	Involved in the intramitochondrial synthesis of acylcarnitines from accumulated acyl-CoA metabolites.<br>Reconverts acylcarnitines back into the respective acyl-CoA esters that can then undergo beta-oxidation, an essential step for the mitochondrial uptake of long-chain fatty acids and their subsequent beta-oxidation in the mitochondrion.
227	SLC25A29	solute carrier family 25 member 29	Transports arginine, lysine, homoarginine, methylarginine and, to a much lesser extent, ornithine and histidine
228	SLC25A20	solute carrier family 25 member 20	Mediates the transport of acylcarnitines of different length across the mitochondrial inner membrane from the cytosol to the mitochondrial matrix for their oxidation by the mitochondrial fatty acid-oxidation pathway.
229	CPT1A	carnitine palmitoyltransferase 1A	Catalyzes the transfer of the acyl group of long-chain fatty acid-CoA conjugates onto carnitine, an essential step for the mitochondrial uptake of long-chain fatty acids and their subsequent beta-oxidation in the mitochondrion
230	CPT1B	carnitine palmitoyltransferase 1B	This protein is produced by a bicistronic gene which also produces the CHKB protein from a non-overlapping reading frame.
231	CPT1C	carnitine palmitoyltransferase 1C	May play a role in lipid metabolic process.

Figure S2. Thermogenesis score and survival characteristics in patients with (A) estrogen receptor (ER)-positive/human epidermal growth factor receptor 2 (Her 2)-negative breast cancer and (B) Her 2-positive breast cancer. Kaplan–Meier survival plots are shown comparing high and low thermogenesis score along with *p*-values for disease-specific survival (DSS), disease-free survival (DFS), and overall survival (OS). The survivals between high and low thermogenesis groups were compared using log-rank tests.

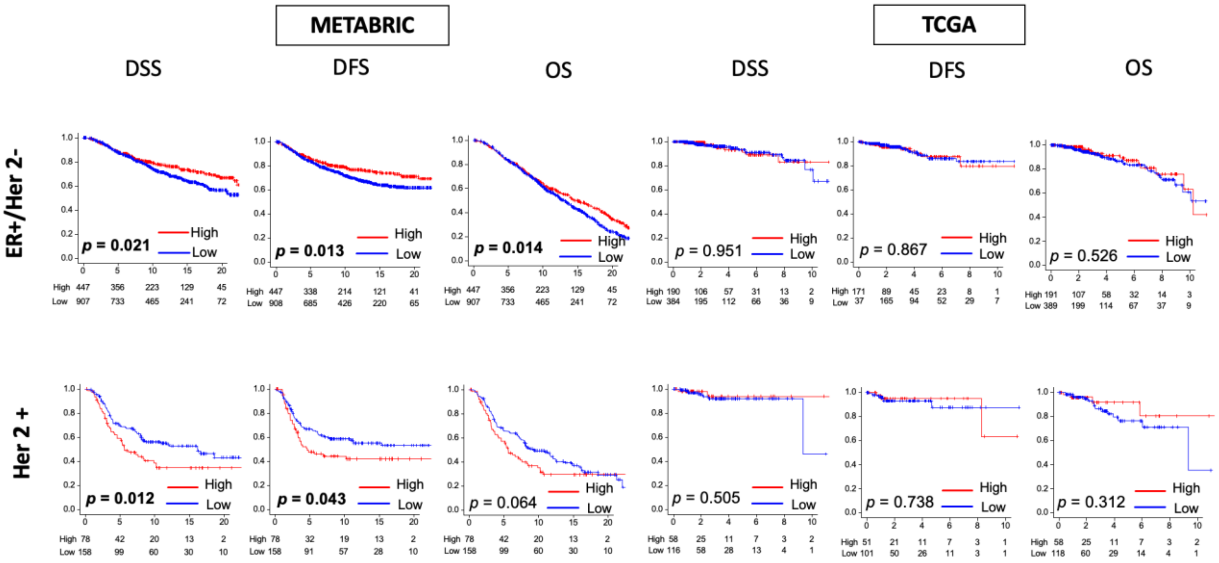

Figure S3. Hallmark gene sets with significant enrichment in triple-negative breast cancer (TNBC) with high and low thermogenesis scores in METABRIC, TCGA, and GSE96058 cohorts. Gene set enrichment analysis (GSEA) plots along with the normalized enrichment score (NES) and false discovery rate (FDR) are shown here for the gene sets where enrichment was seen in high-thermogenesis and low-thermogenesis tumors in METABRIC, TCGA, and GSE96058 cohorts. The cut-off of highest tertile (top 33%) of thermogenesis score was considered as high and the remaining as low. FDR < 0.25 was used to determine statistical significance. Negative NES refers to gene set enrichment in the low thermogenesis group.

METABRIC (298)

High

NAME	NES	NOM p-val	FDR q-val
HALLMARK_FATTY_ACID_METABOLISM	1.835185	0	0
HALLMARK_ADIPOGENESIS	1.807413	0	8.74E-04
HALLMARK_OXIDATIVE_PHOSPHORYLATION	1.809424	0	0.001312
HALLMARK_PROTEIN_SECRETION	1.606299	0.009728	0.038586
HALLMARK_XENOBIOTIC_METABOLISM	1.566795	0.003984	0.04611
HALLMARK_BILE_ACID_METABOLISM	1.552092	0.004073	0.048746
HALLMARK_PANCREAS_BETA_CELLS	1.568484	0.02988	0.05457
HALLMARK_MYOGENESIS	1.520148	0.01848	0.065524
HALLMARK_PEROXISOME	1.448946	0.027888	0.11839
HALLMARK_TGF_BETA_SIGNALING	1.410216	0.077079	0.147816
HALLMARK_UV_RESPONSE_DN	1.391738	0.047917	0.155394
HALLMARK_ANDROGEN_RESPONSE	1.36391	0.060041	0.172606
HALLMARK_CHOLESTEROL_HOMEOSTASIS	1.33984	0.062124	0.175763
HALLMARK_ANGIOGENESIS	1.351005	0.131078	0.1759
HALLMARK_REACTIVE_OXYGEN_SPECIES_PATHWAY	1.314038	0.087209	0.181163
HALLMARK_HEME_METABOLISM	1.314297	0.026119	0.192915

Low

NAME	NES	NOM p-val	FDR q-val
HALLMARK_MITOTIC_SPINDLE	-1.6329824	0.00625	0.061802194
HALLMARK_E2F_TARGETS	-1.3996549	0.11134904	0.16667327
HALLMARK_INTERFERON_ALPHA_RESPONSE	-1.4192331	0.07014028	0.17032547
HALLMARK_ALLOGRAFT_REJECTION	-1.5154049	0.033864543	0.17482497
HALLMARK_INTERFERON_GAMMA_RESPONSE	-1.3611813	0.111788616	0.19027962
HALLMARK_G2M_CHECKPOINT	-1.432581	0.0751073	0.1914344
HALLMARK_MYC_TARGETS_V2	-1.460645	0.08782435	0.19810869

GSE96058 (143)

High

NAME	NES	NOM p-val	FDR q-val
HALLMARK_ADIPOGENESIS	1.99184	0	0.002237
HALLMARK_FATTY_ACID_METABOLISM	1.869096	0.001946	0.007932
HALLMARK_BILE_ACID_METABOLISM	1.698439	0.003891	0.046432
HALLMARK_XENOBIOTIC_METABOLISM	1.621068	0.017893	0.085655
HALLMARK_ANDROGEN_RESPONSE	1.561443	0.03301	0.11554
HALLMARK_PEROXISOME	1.502552	0.02994	0.150846

Low

NAME	NES	NOM p-val	FDR q-val
HALLMARK_MITOTIC_SPINDLE	-1.7515	0	0.101713
HALLMARK_G2M_CHECKPOINT	-1.68383	0	0.113465
HALLMARK_E2F_TARGETS	-1.63383	0.005848	0.128834
HALLMARK_MYC_TARGETS_V1	-1.62629	0.048356	0.104706
HALLMARK_MYC_TARGETS_V2	-1.57197	0.038685	0.142341
HALLMARK_INTERFERON_ALPHA_RESPONSE	-1.569	0.048	0.121813
HALLMARK_INTERFERON_GAMMA_RESPONSE	-1.47864	0.100604	0.215924
HALLMARK_DNA_REPAIR	-1.43909	0.110236	0.24582

Table S3: List of genes included in the hallmark gene sets with significant enrichment.

***HALLMARK_FATTY_ACID_METABOLISM* : Genes encoding proteins involved in fatty acid metabolism.**

Gene Symbol	Gene Description
AADAT	aminoadipate aminotransferase
ACAA1	acetyl-CoA acyltransferase 1
ACAA2	acetyl-CoA acyltransferase 2
ACADL	acyl-CoA dehydrogenase long chain
ACADM	acyl-CoA dehydrogenase medium chain
ACADS	acyl-CoA dehydrogenase short chain
ACADVL	acyl-CoA dehydrogenase very long chain
ACAT2	acetyl-CoA acetyltransferase 2
ACO2	aconitase 2
ACOT2	acyl-CoA thioesterase 2
ACOT8	acyl-CoA thioesterase 8
ACOX1	acyl-CoA oxidase 1
ACSL1	acyl-CoA synthetase long chain family member 1
ACSL4	acyl-CoA synthetase long chain family member 4
ACSL5	acyl-CoA synthetase long chain family member 5
ACSM3	acyl-CoA synthetase medium chain family member 3
ACSS1	acyl-CoA synthetase short chain family member 1
ADH1C	alcohol dehydrogenase 1C (class I), gamma polypeptide
ADH7	alcohol dehydrogenase 7 (class IV), mu or sigma polypeptide
ADIPOR2	adiponectin receptor 2
ADSL	adenylosuccinate lyase
ALAD	aminolevulinate dehydratase
ALDH1A1	aldehyde dehydrogenase 1 family member A1
ALDH3A1	aldehyde dehydrogenase 3 family member A1
ALDH3A2	aldehyde dehydrogenase 3 family member A2
ALDH9A1	aldehyde dehydrogenase 9 family member A1
ALDOA	"aldolase, fructose-bisphosphate A
AOC3	amine oxidase copper containing 3
APEX1	apurinic/apyrimidinic endodeoxyribonuclease 1
AQP7	aquaporin 7
AUH	AU RNA binding methylglutaconyl-CoA hydratase
BCKDHB	branched chain keto acid dehydrogenase E1 subunit beta
BLVRA	biliverdin reductase A

BMPR1B	bone morphogenetic protein receptor type 1B
BPHL	biphenyl hydrolase like
CA2	carbonic anhydrase 2
CA4	carbonic anhydrase 4
CA6	carbonic anhydrase 6
CBR1	carbonyl reductase 1
CBR3	carbonyl reductase 3
CCDC58	coiled-coil domain containing 58
CD1D	CD1d molecule
CD36	CD36 molecule
CEL	carboxyl ester lipase
CIDEA	cell death inducing DFFA like effector A
CPOX	coproporphyrinogen oxidase
CPT1A	carnitine palmitoyltransferase 1A
CPT2	carnitine palmitoyltransferase 2
CRAT	carnitine O-acetyltransferase
CRYZ	crystallin zeta
CYP1A1	cytochrome P450 family 1 subfamily A member 1
CYP4A11	cytochrome P450 family 4 subfamily A member 11
CYP4A22	cytochrome P450 family 4 subfamily A member 22
D2HGDH	D-2-hydroxyglutarate dehydrogenase
DECR1	"2,4-dienoyl-CoA reductase 1
DHCR24	24-dehydrocholesterol reductase
DLD	dihydrolipoamide dehydrogenase
DLST	dihydrolipoamide S-succinyltransferase
ECH1	enoyl-CoA hydratase 1
ECHS1	"enoyl-CoA hydratase, short chain 1
ECI1	enoyl-CoA delta isomerase 1
ECI2	enoyl-CoA delta isomerase 2
EHHADH	enoyl-CoA hydratase and 3-hydroxyacyl CoA dehydrogenase
ELOVL5	ELOVL fatty acid elongase 5
ENO2	enolase 2
ENO3	enolase 3
EPHX1	epoxide hydrolase 1
ERP29	endoplasmic reticulum protein 29
ETFDH	electron transfer flavoprotein dehydrogenase
FABP1	fatty acid binding protein 1
FABP2	fatty acid binding protein 2

FASN	fatty acid synthase
FH	fumarate hydratase
FMO1	flavin containing dimethylaniline monooxygenase 1
G0S2	G0/G1 switch 2
GABARAPL1	GABA type A receptor associated protein like 1
GAD2	glutamate decarboxylase 2
GAPDHS	"glyceraldehyde-3-phosphate dehydrogenase, spermatogenic
GCDH	glutaryl-CoA dehydrogenase
GLUL	glutamate-ammonia ligase
GPD1	glycerol-3-phosphate dehydrogenase 1
GPD2	glycerol-3-phosphate dehydrogenase 2
GRHPR	glyoxylate and hydroxypyruvate reductase
GSTZ1	glutathione S-transferase zeta 1
H2AZ1	H2A.Z variant histone 1
HADH	hydroxyacyl-CoA dehydrogenase
HADHB	hydroxyacyl-CoA dehydrogenase trifunctional multienzyme complex subunit beta
HAO2	hydroxyacid oxidase 2
HCCS	holocytochrome c synthase
HIBCH	3-hydroxyisobutyryl-CoA hydrolase
HMGCL	3-hydroxy-3-methylglutaryl-CoA lyase
HMGCS1	3-hydroxy-3-methylglutaryl-CoA synthase 1
HMGCS2	3-hydroxy-3-methylglutaryl-CoA synthase 2
HPGD	15-hydroxyprostaglandin dehydrogenase
HSD17B10	hydroxysteroid 17-beta dehydrogenase 10
HSD17B11	hydroxysteroid 17-beta dehydrogenase 11
HSD17B4	hydroxysteroid 17-beta dehydrogenase 4
HSD17B7	hydroxysteroid 17-beta dehydrogenase 7
HSDL2	hydroxysteroid dehydrogenase like 2
HSP90AA1	heat shock protein 90 alpha family class A member 1
HSPH1	heat shock protein family H (Hsp110) member 1
IDH1	isocitrate dehydrogenase (NADP(+)) 1
IDH3B	isocitrate dehydrogenase (NAD(+)) 3 non catalytic subunit beta
IDH3G	isocitrate dehydrogenase (NAD(+)) 3 non-catalytic subunit gamma
IDI1	isopentenyl-diphosphate delta isomerase 1
IL4I1	interleukin 4 induced 1
INMT	indolethylamine N-methyltransferase
LDHA	lactate dehydrogenase A
LGALS1	galectin 1

LTC4S	leukotriene C4 synthase
MAOA	monoamine oxidase A
MCEE	methylmalonyl-CoA epimerase
MDH1	malate dehydrogenase 1
MDH2	malate dehydrogenase 2
ME1	malic enzyme 1
METAP1	methionyl aminopeptidase 1
MGLL	monoglyceride lipase
MIF	macrophage migration inhibitory factor
MLYCD	malonyl-CoA decarboxylase
NBN	nibrin
NCAPH2	non-SMC condensin II complex subunit H2
NSDHL	NAD(P) dependent steroid dehydrogenase-like
NTHL1	nth like DNA glycosylase 1
ODC1	ornithine decarboxylase 1
OSTC	oligosaccharyltransferase complex non-catalytic subunit
PCBD1	pterin-4 alpha-carbinolamine dehydratase 1
PDHA1	pyruvate dehydrogenase E1 subunit alpha 1
PDHB	pyruvate dehydrogenase E1 subunit beta
PPARA	peroxisome proliferator activated receptor alpha
PRDX6	peroxiredoxin 6
PSME1	proteasome activator subunit 1
PTPRG	protein tyrosine phosphatase receptor type G
PTS	6-pyruvoyltetrahydropterin synthase
RAP1GDS1	Rap1 GTPase-GDP dissociation stimulator 1
RDH11	retinol dehydrogenase 11
RDH16	retinol dehydrogenase 16
REEP6	receptor accessory protein 6
RETSAT	retinol saturase
S100A10	S100 calcium binding protein A10
SDHA	succinate dehydrogenase complex flavoprotein subunit A
SDHC	succinate dehydrogenase complex subunit C
SDHD	succinate dehydrogenase complex subunit D
SERINC1	serine incorporator 1
KMT5A	lysine methyltransferase 5A
SLC22A5	solute carrier family 22 member 5
SMS	spermine synthase
SUCLA2	succinate-CoA ligase ADP-forming subunit beta

SUCLG1	succinate-CoA ligase GDP/ADP-forming subunit alpha
SUCLG2	succinate-CoA ligase GDP-forming subunit beta
TDO2	"tryptophan 2,3-dioxygenase
TP53INP2	tumor protein p53 inducible nuclear protein 2
UBE2L6	ubiquitin conjugating enzyme E2 L6
UGDH	UDP-glucose 6-dehydrogenase
UROD	uroporphyrinogen decarboxylase
UROS	uroporphyrinogen III synthase
VNN1	vanin 1
XIST	X inactive specific transcript
YWHAH	tyrosine 3-monooxygenase/tryptophan 5-monooxygenase activation protein eta

HALLMARK_ADIPOGENESIS : Genes up-regulated during adipocyte differentiation (adipogenesis)

**Gene Symbol**	**Gene Description**
ABCA1	ATP binding cassette subfamily A member 1
ABCB8	ATP binding cassette subfamily B member 8
ACAA2	acetyl-CoA acyltransferase 2
ACADL	acyl-CoA dehydrogenase long chain
ACADM	acyl-CoA dehydrogenase medium chain
ACADS	acyl-CoA dehydrogenase short chain
ACLY	ATP citrate lyase
ACO2	aconitase 2
ACOX1	acyl-CoA oxidase 1
ADCY6	adenylate cyclase 6
ADIG	adipogenin
ADIPOQ	adiponectin, C1Q and collagen domain containing
ADIPOR2	adiponectin receptor 2
AGPAT3	1-acylglycerol-3-phosphate O-acyltransferase 3
GPAT4	glycerol-3-phosphate acyltransferase 4
AIFM1	apoptosis inducing factor mitochondria associated 1
AK2	adenylate kinase 2
ALDH2	aldehyde dehydrogenase 2 family member
ALDOA	aldolase, fructose-bisphosphate A
ANGPT1	angiopoietin 1
ANGPTL4	angiopoietin like 4
SOWAHC	sosondowah ankyrin repeat domain family member C

APLP2	amyloid beta precursor like protein 2
APOE	apolipoprotein E
ARAF	A-Raf proto-oncogene, serine/threonine kinase
ARL4A	ADP ribosylation factor like GTPase 4A
ATL2	atlastin GTPase 2
ATP1B3	ATPase Na+/K+ transporting subunit beta 3
ATP5PO	ATP synthase peripheral stalk subunit OSCP
BAZ2A	bromodomain adjacent to zinc finger domain 2A
BCKDHA	branched chain keto acid dehydrogenase E1 subunit alpha
BCL2L13	BCL2 like 13
BCL6	BCL6 transcription repressor
C3	complement C3
CAT	catalase
CCNG2	cyclin G2
CD151	CD151 molecule (Raph blood group)
CD302	CD302 molecule
CD36	CD36 molecule
CDKN2C	cyclin dependent kinase inhibitor 2C
CHCHD10	coiled-coil-helix-coiled-coil-helix domain containing 10
CHUK	component of inhibitor of nuclear factor kappa B kinase complex
CIDEA	cell death inducing DFFA like effector a
CMBL	carboxymethylenebutenolidase homolog
CMPK1	cytidine/uridine monophosphate kinase 1
COL15A1	collagen type XV alpha 1 chain
COL4A1	collagen type IV alpha 1 chain
COQ3	coenzyme Q3, methyltransferase
COQ5	coenzyme Q5, methyltransferase
COQ9	coenzyme Q9
COX6A1	cytochrome c oxidase subunit 6A1
COX7B	cytochrome c oxidase subunit 7B
COX8A	cytochrome c oxidase subunit 8A
CPT2	carnitine palmitoyltransferase 2
CRAT	carnitine O-acetyltransferase
CS	citrate synthase
CYC1	cytochrome c1
CYP4B1	cytochrome P450 family 4 subfamily B member 1
DBT	dihydrolipoamide branched chain transacylase E2
DDT	D-dopachrome tautomerase

DECR1	2,4-dienoyl-CoA reductase 1
DGAT1	diacylglycerol O-acyltransferase 1
DHCR7	7-dehydrocholesterol reductase
DHRS7	dehydrogenase/reductase 7
DHRS7B	dehydrogenase/reductase 7B
DLAT	dihydrolipoamide S-acetyltransferase
DLD	dihydrolipoamide dehydrogenase
DNAJB9	DnaJ heat shock protein family (Hsp40) member B9
DNAJC15	DnaJ heat shock protein family (Hsp40) member C15
DRAM2	DNA damage regulated autophagy modulator 2
ECH1	enoyl-CoA hydratase 1
ECHS1	enoyl-CoA hydratase, short chain 1
ELMOD3	ELMO domain containing 3
ELOVL6	ELOVL fatty acid elongase 6
ENPP2	ectonucleotide pyrophosphatase/phosphodiesterase 2
EPHX2	epoxide hydrolase 2
ESRRA	estrogen related receptor alpha
ESYT1	extended synaptotagmin 1
ETFB	electron transfer flavoprotein subunit beta
FABP4	fatty acid binding protein 4
FAH	fumarylacetoacetate hydrolase
MIGA2	mitoguardin 2
RMDN3	regulator of microtubule dynamics 3
FZD4	frizzled class receptor 4
G3BP2	G3BP stress granule assembly factor 2
GADD45A	growth arrest and DNA damage inducible alpha
GBE1	1,4-alpha-glucan branching enzyme 1
GHITM	growth hormone inducible transmembrane protein
GPAM	"glycerol-3-phosphate acyltransferase, mitochondrial
GPD2	glycerol-3-phosphate dehydrogenase 2
GPHN	gephyrin
GPX3	glutathione peroxidase 3
GPX4	glutathione peroxidase 4
GRPEL1	GrpE like 1, mitochondrial
HADH	hydroxyacyl-CoA dehydrogenase
HIBCH	3-hydroxyisobutyryl-CoA hydrolase
HSPB8	heat shock protein family B (small) member 8
IDH1	isocitrate dehydrogenase (NADP(+)) 1

IDH3A	isocitrate dehydrogenase (NAD(+)) 3 catalytic subunit alpha
IDH3G	isocitrate dehydrogenase (NAD(+)) 3 non-catalytic subunit gamma
IFNGR1	interferon gamma receptor 1
IMMT	inner membrane mitochondrial protein
ITGA7	integrin subunit alpha 7
ITIH5	inter-alpha-trypsin inhibitor heavy chain 5
ITSN1	intersectin 1
JAGN1	jagunal homolog 1
LAMA4	laminin subunit alpha 4
LEP	leptin
LIFR	LIF receptor subunit alpha
LIPE	lipase E, hormone sensitive type
LPCAT3	lysophosphatidylcholine acyltransferase 3
LPL	lipoprotein lipase
LTC4S	leukotriene C4 synthase
MAP4K3	mitogen-activated protein kinase kinase kinase 3
MCCC1	methylcrotonoyl-CoA carboxylase 1
MDH2	malate dehydrogenase 2
ME1	malic enzyme 1
MGLL	monoglyceride lipase
MGST3	microsomal glutathione S-transferase 3
MTARC2	mitochondrial amidoxime reducing component 2
MRAP	melanocortin 2 receptor accessory protein
MRPL15	mitochondrial ribosomal protein L15
MTCH2	mitochondrial carrier 2
MYLK	myosin light chain kinase
NDUFA5	NADH:ubiquinone oxidoreductase subunit A5
NDUFAB1	NADH:ubiquinone oxidoreductase subunit AB1
NDUFB7	NADH:ubiquinone oxidoreductase subunit B7
NDUFS3	NADH:ubiquinone oxidoreductase core subunit S3
NKIRAS1	NFKB inhibitor interacting Ras like 1
NMT1	N-myristoyltransferase 1
NABP1	nucleic acid binding protein 1
OMD	osteomodulin
ORM1	orosomucoid 1
PDCD4	programmed cell death 4
PEMT	phosphatidylethanolamine N-methyltransferase
PEX14	peroxisomal biogenesis factor 14

PFKFB3	6-phosphofructo-2-kinase/fructose-2,6-biphosphatase 3
PFKL	phosphofructokinase, liver type
PGM1	phosphoglucomutase 1
PHLDB1	pleckstrin homology like domain family B member 1
PHYH	phytanoyl-CoA 2-hydroxylase
PIM3	Pim-3 proto-oncogene, serine/threonine kinase
PLIN2	perilipin 2
POR	cytochrome p450 oxidoreductase
PPARG	peroxisome proliferator activated receptor gamma
PPM1B	protein phosphatase, Mg2+/Mn2+ dependent 1B
PPP1R15B	protein phosphatase 1 regulatory subunit 15B
SLC66A3	solute carrier family 66 member 3
PRDX3	peroxiredoxin 3
PREB	prolactin regulatory element binding
PTCD3	pentatricopeptide repeat domain 3
PTGER3	prostaglandin E receptor 3
CAVIN1	caveolae associated protein 1
QDPR	quinoid dihydropteridine reductase
RAB34	RAB34, member RAS oncogene family
REEP5	receptor accessory protein 5
REEP6	receptor accessory protein 6
RETN	resistin
RETSAT	retinol saturase
RIOK3	RIO kinase 3
RNF11	ring finger protein 11
RREB1	ras responsive element binding protein 1
RTN3	reticulon 3
SAMM50	SAMM50 sorting and assembly machinery component
SCARB1	scavenger receptor class B member 1
SCP2	sterol carrier protein 2
SDHB	succinate dehydrogenase complex iron sulfur subunit B
SDHC	succinate dehydrogenase complex subunit C
CAVIN2	caveolae associated protein 2
SLC19A1	solute carrier family 19 member 1
SLC1A5	solute carrier family 1 member 5
SLC25A1	solute carrier family 25 member 1
SLC25A10	solute carrier family 25 member 10
SLC27A1	solute carrier family 27 member 1

SLC5A6	solute carrier family 5 member 6
SNCG	synuclein gamma
SOD1	superoxide dismutase 1
SORBS1	sorbin and SH3 domain containing 1
SPARCL1	SPARC like 1
SQOR	sulfide quinone oxidoreductase
SSPN	sarcospan
STAT5A	signal transducer and activator of transcription 5A
STOM	stomatin
SUCLG1	succinate-CoA ligase GDP/ADP-forming subunit alpha
SULT1A1	sulfotransferase family 1A member 1
TALDO1	transaldolase 1
TANK	TRAF family member associated NFKB activator
TKT	transketolase
TOB1	transducer of ERBB2, 1
TST	thiosulfate sulfurtransferase
UBC	ubiquitin C
UBQLN1	ubiquilin 1
UCK1	uridine-cytidine kinase 1
UCP2	uncoupling protein 2
UQCR10	ubiquinol-cytochrome c reductase, complex III subunit X
UQCR11	ubiquinol-cytochrome c reductase, complex III subunit XI
UQCRC1	ubiquinol-cytochrome c reductase core protein 1
UQCRQ	ubiquinol-cytochrome c reductase complex III subunit VII
VEGFB	vascular endothelial growth factor B
YWHAG	tyrosine 3-monooxygenase/tryptophan 5-monooxygenase activation protein gamma

HALLMARK_MITOTIC_SPINDLE: Genes important for mitotic spindle assembly

**Gene Symbol**	**Gene Description**
ABI1	abl interactor 1
ABL1	ABL proto-oncogene 1, non-receptor tyrosine kinase
ABR	ABR activator of RhoGEF and GTPase
ACTN4	actinin alpha 4
AKAP13	A-kinase anchoring protein 13
ALMS1	ALMS1 centrosome and basal body associated protein
ALS2	alsin Rho guanine nucleotide exchange factor ALS2
ANLN	anillin actin binding protein
APC	APC regulator of WNT signaling pathway
ARAP3	ArfGAP with RhoGAP domain, ankyrin repeat and PH domain 3
ARF6	ADP ribosylation factor 6
ARFGEF1	ADP ribosylation factor guanine nucleotide exchange factor 1
ARFIP2	ADP ribosylation factor interacting protein 2
ARHGAP10	Rho GTPase activating protein 10
ARHGAP27	Rho GTPase activating protein 27
ARHGAP29	Rho GTPase activating protein 29
ARHGAP4	Rho GTPase activating protein 4
ARHGAP5	Rho GTPase activating protein 5
ARHGDIA	Rho GDP dissociation inhibitor alpha
ARHGEF11	Rho guanine nucleotide exchange factor 10
ARHGEF12	Rho guanine nucleotide exchange factor 12
ARHGEF2	Rho/Rac guanine nucleotide exchange factor 2
ARHGEF3	Rho guanine nucleotide exchange factor 3
ARHGEF7	Rho guanine nucleotide exchange factor 7
ARL8A	ADP ribosylation factor like GTPase 8A
ATG4B	autophagy related 4B cysteine peptidase
AURKA	aurora kinase A
CEP131	centrosomal protein 131
BCAR1	BCAR1 scaffold protein, Cas family member
BCL2L11	BCL2 like 11
BCR	BCR activator of RhoGEF and GTPase
BIN1	bridging integrator 1
BIRC5	baculoviral IAP repeat containing 5
BRCA2	BRCA2 DNA repair associated
BUB1	BUB1 mitotic checkpoint serine/threonine kinase

CAPZB	capping actin protein of muscle Z-line subunit beta
CCDC88A	coiled-coil domain containing 88A
CCNB2	cyclin B2
CD2AP	CD2 associated protein
CDC27	cell division cycle 27
CDC42	cell division cycle 42
CDC42BPA	CDC42 binding protein kinase alpha
CDC42EP1	CDC42 effector protein 1
CDC42EP2	CDC42 effector protein 2
CDC42EP4	CDC42 effector protein 4
CDK1	cyclin dependent kinase 1
CDK5RAP2	CDK5 regulatory subunit associated protein 2
CENPE	centromere protein E
CENPF	centromere protein F
CENPJ	centromere protein J
CEP192	centrosomal protein 192
CEP250	centrosomal protein 250
CEP57	centrosomal protein 57
CEP72	centrosomal protein 72
CKAP5	cytoskeleton associated protein 5
CLASP1	cytoplasmic linker associated protein 1
CLIP1	CAP-Gly domain containing linker protein 1
CLIP2	CAP-Gly domain containing linker protein 2
CNTRL	centriolin
CNTROB	centrobin, centriole duplication and spindle assembly protein
CRIPAK	
CSNK1D	casein kinase 1 delta
CTTN	cortactin
CYTH2	cytohesin 2
DLG1	discs large MAGUK scaffold protein 1
DLGAP5	DLG associated protein 5
DOCK2	dedicator of cytokinesis 2
DOCK4	dedicator of cytokinesis 4
DST	dystonin
DYNC1H1	dynein cytoplasmic 1 heavy chain 1
DYNLL2	dynein light chain LC8-type 2
ECT2	epithelial cell transforming 2 [
EPB41	erythrocyte membrane protein band 4.1

EPB41L2	erythrocyte membrane protein band 4.1 like 2
ESPL1	extra spindle pole bodies like 1, separase
EZR	ezrin
FARP1	FERM, ARH/RhoGEF and pleckstrin domain protein 1
FBXO5	F-box protein 5
FGD4	FYVE, RhoGEF and PH domain containing 4
FGD6	FYVE, RhoGEF and PH domain containing 6
FLNA	filamin A
FLNB	filamin B
FSCN1	fascin actin-bundling protein 1
GEMIN4	gem nuclear organelle associated protein 4
GSN	gelsolin
HDAC6	histone deacetylase 6
HOOK3	hook microtubule tethering protein 3
INCENP	inner centromere protein
ITSN1	intersectin 1
KATNA1	katanin catalytic subunit A1
KATNB1	katanin regulatory subunit B1
KIF11	kinesin family member 11
KIF15	kinesin family member 15
KIF1B	kinesin family member 1B
KIF20B	kinesin family member 20B
KIF22	kinesin family member 22
KIF23	kinesin family member 23
KIF2C	kinesin family member 2C
KIF3B	kinesin family member 3B
KIF3C	kinesin family member 3C
KIF4A	kinesin family member 4A
KIF5B	kinesin family member 5B
KIFAP3	kinesin associated protein 3
KLC1	kinesin light chain 1
KNTC1	kinetochore associated 1
KPTN	"kaptin, actin binding protein
LATS1	large tumor suppressor kinase 1
LLGL1	LLGL scribble cell polarity complex component 1
LMNB1	lamin B1
LRPPRC	leucine rich pentatricopeptide repeat containing
MAP1S	microtubule associated protein 1S

MAP3K11	mitogen-activated protein kinase kinase kinase 11
MAPRE1	microtubule associated protein RP/EB family member 1
MARCKS	myristoylated alanine rich protein kinase C substrate
MARK4	microtubule affinity regulating kinase 4
MID1	midline 1
MID1IP1	MID1 interacting protein 1
MYH10	myosin heavy chain 10
MYH9	myosin heavy chain 9
MYO1E	myosin IE
MYO9B	myosin IXB
NCK1	NCK adaptor protein 1
NCK2	NCK adaptor protein 2
NDC80	NDC80 kinetochore complex component
NEDD9	neural precursor cell expressed, developmentally down-regulated 9
NEK2	NIMA related kinase 2
NET1	neuroepithelial cell transforming 1
NF1	neurofibromin 1
NIN	ninein
NOTCH2	notch receptor 2
NUMA1	nuclear mitotic apparatus protein 1
NUSAP1	nucleolar and spindle associated protein 1
OPHN1	oligophrenin 1
PAFAH1B1	platelet activating factor acetylhydrolase 1b regulatory subunit 1
PALLD	palladin, cytoskeletal associated protein
PCGF5	polycomb group ring finger 5
PCM1	pericentriolar material 1
PCNT	pericentrin
PDLIM5	PDZ and LIM domain 5
PIF1	PIF1 5'-to-3' DNA helicase
PKD2	polycystin 2, transient receptor potential cation channel
PLEKHG2	pleckstrin homology and RhoGEF domain containing G2
PLK1	polo like kinase 1
PPP4R2	protein phosphatase 4 regulatory subunit 2
PRC1	protein regulator of cytokinesis 1
PREX1	phosphatidylinositol-3,4,5-trisphosphate dependent Rac exchange factor 1
PXN	paxillin
RAB3GAP1	RAB3 GTPase activating protein catalytic subunit 1
RABGAP1	RAB GTPase activating protein 1

RACGAP1	Rac GTPase activating protein 1
RALBP1	ralA binding protein 1
RANBP9	RAN binding protein 9
RAPGEF5	Rap guanine nucleotide exchange factor 5
RAPGEF6	Rap guanine nucleotide exchange factor 6
RASA1	RAS p21 protein activator 1
RASA2	RAS p21 protein activator 2
RASAL2	RAS protein activator like 2
RFC1	replication factor C subunit 1
RHOF	ras homolog family member F, filopodia associated
RHOT2	ras homolog family member T2
RICTOR	RPTOR independent companion of MTOR complex 2
ROCK1	Rho associated coiled-coil containing protein kinase 1
SAC3D1	SAC3 domain containing 1
SASS6	SAS-6 centriolar assembly protein
SEPTIN9	septin 9
SHROOM1	shroom family member 1
SHROOM2	shroom family member 2
SMC1A	structural maintenance of chromosomes 1A
SMC3	structural maintenance of chromosomes 3
SMC4	structural maintenance of chromosomes 4
SORBS2	sorbin and SH3 domain containing 2
SOS1	SOS Ras/Rac guanine nucleotide exchange factor 1
SPTAN1	spectrin alpha, non-erythrocytic 1
SPTBN1	spectrin beta, non-erythrocytic 1
SSH2	slingshot protein phosphatase 2
STAU1	staufen double-stranded RNA binding protein 1
STK38L	serine/threonine kinase 38 like
SUN2	Sad1 and UNC84 domain containing 2
SYNPO	synaptopodin
TAOK2	TAO kinase 2
TBCD	tubulin folding cofactor D
TIAM1	TIAM Rac1 associated GEF 1
TLK1	tousled like kinase 1
TOP2A	DNA topoisomerase II alpha
TPX2	TPX2 microtubule nucleation factor
TRIO	trio Rho guanine nucleotide exchange factor
TSC1	TSC complex subunit 1

TTK	TTK protein kinase
TUBA4A	tubulin alpha 4a
TUBD1	tubulin delta 1
TUBGCP2	tubulin gamma complex associated protein 2
TUBGCP3	tubulin gamma complex associated protein 3
TUBGCP5	tubulin gamma complex associated protein 5
TUBGCP6	tubulin gamma complex associated protein 6
UXT	ubiquitously expressed prefoldin like chaperone
VCL	vinculin
WASF1	WASP family member 1
WASF2	WASP family member 2
WASL	WASP like actin nucleation promoting factor
YWHAE	tyrosine 3-monooxygenase/tryptophan 5-monooxygenase activation protein epsilon

HALLMARK_E2F_TARGETS: Genes encoding cell-cycle-related targets of E2F transcription factors

**Gene Symbol**	**Gene Description**
AK2	adenylate kinase 2
ANP32E	acidic nuclear phosphoprotein 32 family member E
ASF1A	anti-silencing function 1A histone chaperone
ASF1B	anti-silencing function 1B histone chaperone
ATAD2	ATPase family AAA domain containing 2
AURKA	aurora kinase A
AURKB	aurora kinase B
BARD1	BRCA1 associated RING domain 1
BIRC5	baculoviral IAP repeat containing 5
BRCA1	BRCA1 DNA repair associated
BRCA2	BRCA2 DNA repair associated
BRMS1L	BRMS1 like transcriptional repressor
BUB1B	BUB1 mitotic checkpoint serine/threonine kinase B
CBX5	chromobox 5
CCNB2	cyclin B2
CCNE1	cyclin E1
CCP110	centriolar coiled-coil protein 110
CDC20	cell division cycle 20
CDC25A	cell division cycle 25A
CDC25B	cell division cycle 25B
CDCA3	cell division cycle associated 3
CDCA8	cell division cycle associated 8
CDK1	cyclin dependent kinase 1
CDK4	cyclin dependent kinase 4
CDKN1A	cyclin dependent kinase inhibitor 1A
CDKN1B	cyclin dependent kinase inhibitor 1B
CDKN2A	cyclin dependent kinase inhibitor 2A
CDKN2C	cyclin dependent kinase inhibitor 2C
CDKN3	cyclin dependent kinase inhibitor 3
CENPE	centromere protein E
CENPM	centromere protein M
CHEK1	checkpoint kinase 1
CHEK2	checkpoint kinase 2
CIT	citron rho-interacting serine/threonine kinase
CKS1B	CDC28 protein kinase regulatory subunit 1B

CKS2	CDC28 protein kinase regulatory subunit 2
CSE1L	chromosome segregation 1 like
CTCF	CCCTC-binding factor
CTPS1	CTP synthase 1
DCK	deoxycytidine kinase
DCLRE1B	DNA cross-link repair 1B
DCTPP1	dCTP pyrophosphatase 1
DDX39A	DExD-box helicase 39A
DEK	DEK proto-oncogene
DEPDC1	DEP domain containing 1
DIAPH3	diaphanous related formin 3
DLGAP5	DLG associated protein 5
DNMT1	DNA methyltransferase 1
DONSON	DNA replication fork stabilization factor DONSON
DSCC1	DNA replication and sister chromatid cohesion 1
DUT	deoxyuridine triphosphatase
E2F8	E2F transcription factor 8
EED	embryonic ectoderm development
EIF2S1	eukaryotic translation initiation factor 2 subunit alpha
ESPL1	extra spindle pole bodies like 1, separase
EXOSC8	exosome component 8
EZH2	enhancer of zeste 2 polycomb repressive complex 2 subunit
GINS1	GINS complex subunit 1
GINS3	GINS complex subunit 3
GINS4	GINS complex subunit 4
GSPT1	G1 to S phase transition 1
H2AX	H2A.X variant histone
H2AZ1	H2A.Z variant histone 1
HELLS	helicase, lymphoid specific
HMGA1	high mobility group AT-hook 1
HMGB2	high mobility group box 2
HMGB3	high mobility group box 3
HMMR	hyaluronan mediated motility receptor
JPT1	Jupiter microtubule associated homolog 1
HNRNPD	heterogeneous nuclear ribonucleoprotein D
HUS1	HUS1 checkpoint clamp component
ILF3	interleukin enhancer binding factor 3
ING3	inhibitor of growth family member 3

IPO7	importin 7
KIF18B	kinesin family member 18B
KIF22	kinesin family member 22
KIF2C	kinesin family member 2C
KIF4A	kinesin family member 4A
KPNA2	karyopherin subunit alpha 2
LBR	lamin B receptor
LIG1	DNA ligase 1
LMNB1	lamin B1
LUC7L3	LUC7 like 3 pre-mRNA splicing factor
LYAR	Ly1 antibody reactive
MAD2L1	mitotic arrest deficient 2 like 1
MCM2	minichromosome maintenance complex component 2
MCM3	minichromosome maintenance complex component 3
MCM4	minichromosome maintenance complex component 4
MCM5	minichromosome maintenance complex component 5
MCM6	minichromosome maintenance complex component 6
MCM7	minichromosome maintenance complex component 7
MELK	maternal embryonic leucine zipper kinase
MKI67	marker of proliferation Ki-67
MLH1	mutL homolog 1
MMS22L	MMS22 like, DNA repair protein
MRE11	MRE11 homolog, double strand break repair nuclease
MSH2	mutS homolog 2
MTHFD2	methylenetetrahydrofolate dehydrogenase (NADP+dependent)2, methenyltetrahydrofolate cyclohydrolase
MXD3	MAX dimerization protein 3
MYBL2	MYB proto-oncogene like 2
MYC	MYC proto-oncogene, bHLH transcription factor
NAA38	N-alpha-acetyltransferase 38, NatC auxiliary subunit
NAP1L1	nucleosome assembly protein 1 like 1
NASP	nuclear autoantigenic sperm protein
NBN	nibrin
NCAPD2	non-SMC condensin I complex subunit D2
NME1	NME/NM23 nucleoside diphosphate kinase 1
NOLC1	nucleolar and coiled-body phosphoprotein 1
NOP56	NOP56 ribonucleoprotein
NUDT21	nudix hydrolase 21

NUP107	nucleoporin 107
NUP153	nucleoporin 153
NUP205	nucleoporin 205
ORC2	origin recognition complex subunit 2
ORC6	origin recognition complex subunit 6
PA2G4	proliferation-associated 2G4
PAICS	phosphoribosylaminoimidazole carboxylase and phosphoribosylaminoimidazolesuccinocarboxamide synthase
PAN2	poly(A) specific ribonuclease subunit PAN2
PCNA	proliferating cell nuclear antigen
PDS5B	PDS5 cohesin associated factor B
PHF5A	PHD finger protein 5A
PLK1	polo like kinase 1
PLK4	polo like kinase 4
PMS2	PMS1 homolog 2, mismatch repair system component
PNN	pinin, desmosome associated protein
POLA2	DNA polymerase alpha 2, accessory subunit
POLD1	DNA polymerase delta 1, catalytic subunit
POLD2	DNA polymerase delta 2, accessory subunit
POLD3	DNA polymerase delta 3, accessory subunit
POLE	DNA polymerase epsilon, catalytic subunit
POLE4	DNA polymerase epsilon 4, accessory subunit
POP7	POP7 homolog, ribonuclease P/MRP subunit
PPM1D	protein phosphatase, Mg2+/Mn2+ dependent 1D
PPP1R8	protein phosphatase 1 regulatory subunit 8
PRDX4	peroxiredoxin 4
PRIM2	DNA primase subunit 2
PRKDC	protein kinase, DNA-activated, catalytic subunit
PRPS1	phosphoribosyl pyrophosphate synthetase 1
PSIP1	PC4 and SFRS1 interacting protein 1
PSMC3IP	PSMC3 interacting protein
PTTG1	PTTG1 regulator of sister chromatid separation, securin
RACGAP1	Rac GTPase activating protein 1
RAD1	RAD1 checkpoint DNA exonuclease
RAD21	RAD21 cohesin complex component
RAD50	RAD50 double strand break repair protein
RAD51AP1	RAD51 associated protein 1
RAD51C	RAD51 paralog C

RAN	RAN, member RAS oncogene family
RANBP1	RAN binding protein 1
RBBP7	RB binding protein 7, chromatin remodeling factor
RFC1	replication factor C subunit 1
RFC2	replication factor C subunit 2
RFC3	replication factor C subunit 3
RNASEH2A	ribonuclease H2 subunit A
RPA1	replication protein A1
RPA2	replication protein A2
RPA3	replication protein A3
CNOT9	CCR4-NOT transcription complex subunit 9
RRM2	ribonucleotide reductase regulatory subunit M2
SHMT1	serine hydroxymethyltransferase 1
SLBP	stem-loop binding protein
SMC1A	structural maintenance of chromosomes 1A
SMC3	structural maintenance of chromosomes 3
SMC4	structural maintenance of chromosomes 4
SMC6	structural maintenance of chromosomes 6
SNRPB	small nuclear ribonucleoprotein polypeptides B and B1
SPAG5	sperm associated antigen 5
SPC24	SPC24 component of NDC80 kinetochore complex
SPC25	SPC25 component of NDC80 kinetochore complex
SRSF1	serine and arginine rich splicing factor 1
SRSF2	serine and arginine rich splicing factor 2
SSRP1	structure specific recognition protein 1
STAG1	stromal antigen 1
STMN1	stathmin 1
SUV39H1	suppressor of variegation 3-9 homolog 1
SYNCRIP	synaptotagmin binding cytoplasmic RNA interacting protein
TACC3	transforming acidic coiled-coil containing protein 3
TBRG4	transforming growth factor beta regulator 4
TCF19	transcription factor 19
TFRC	transferrin receptor
TIMELESS	timeless circadian regulator
TIPIN	TIMELESS interacting protein
TK1	thymidine kinase 1
TMPO	thymopoietin
TOP2A	DNA topoisomerase II alpha

TP53	tumor protein p53
TRA2B	transformer 2 beta homolog
TRIP13	thyroid hormone receptor interactor 13
TUBB	tubulin beta class I
TUBG1	tubulin gamma 1
UBE2S	ubiquitin conjugating enzyme E2 S
UBE2T	ubiquitin conjugating enzyme E2 T
UBR7	ubiquitin protein ligase E3 component n-recognin 7
UNG	uracil DNA glycosylase
USP1	ubiquitin specific peptidase 1
WDR90	WD repeat domain 90
WEE1	WEE1 G2 checkpoint kinase
XPO1	exportin 1
XRCC6	X-ray repair cross complementing 6
ZW10	zw10 kinetochore protein

HALLMARK_INFLAMMATORY_ALPHA_RESPONSE: Genes up-regulated in response to alpha interferon proteins

**Gene Symbol**	**Gene Description**
ADAR	adenosine deaminase RNA specific
B2M	beta-2-microglobulin
BATF2	basic leucine zipper ATF-like transcription factor 2
BST2	bone marrow stromal cell antigen 2
C1S	complement C1s
CASP1	caspase 1
CASP8	caspase 8
CCRL2	C-C motif chemokine receptor like 2
CD47	CD47 molecule
CD74	CD74 molecule
CMPK2	cytidine/uridine monophosphate kinase 2
CNP	2',3'-cyclic nucleotide 3' phosphodiesterase
CSF1	colony stimulating factor 1
CXCL10	C-X-C motif chemokine ligand 10
CXCL11	C-X-C motif chemokine ligand 11
DDX60	DExD/H-box helicase 60
DHX58	DExH-box helicase 58
EIF2AK2	eukaryotic translation initiation factor 2 alpha kinase 2
ELF1	E74 like ETS transcription factor 1
EPSTI1	epithelial stromal interaction 1
MVB12A	multivesicular body subunit 12A
TENT5A	terminal nucleotidyltransferase 5A
CMTR1	cap methyltransferase 1
GBP2	guanylate binding protein 2
GBP4	guanylate binding protein 4
GMPR	guanosine monophosphate reductase
HERC6	HECT and RLD domain containing E3 ubiquitin protein ligase family member 6
HLA-C	major histocompatibility complex, class I, C
IFI27	interferon alpha inducible protein 27
IFI30	IFI30 lysosomal thiol reductase
IFI35	interferon induced protein 35
IFI44	interferon induced protein 44
IFI44L	interferon induced protein 44 like
IFIH1	interferon induced with helicase C domain 1

IFIT2	interferon induced protein with tetratricopeptide repeats 2
IFIT3	interferon induced protein with tetratricopeptide repeats 3
IFITM1	interferon induced transmembrane protein 1
IFITM2	interferon induced transmembrane protein 2
IFITM3	interferon induced transmembrane protein 3
IL15	interleukin 15
IL4R	interleukin 4 receptor
IL7	interleukin 7
IRF1	interferon regulatory factor 1
IRF2	interferon regulatory factor 2
IRF7	interferon regulatory factor 7
IRF9	interferon regulatory factor 9
ISG15	ISG15 ubiquitin like modifier
ISG20	interferon stimulated exonuclease gene 20
LAMP3	lysosomal associated membrane protein 3
LAP3	leucine aminopeptidase 3
LGALS3BP	galectin 3 binding protein
LPAR6	lysophosphatidic acid receptor 6
LY6E	lymphocyte antigen 6 family member E
MOV10	Mov10 RISC complex RNA helicase
MX1	MX dynamin like GTPase 1
NCOA7	nuclear receptor coactivator 7
NMI	N-myc and STAT interactor
NUB1	negative regulator of ubiquitin like proteins 1
OAS1	2'-5'-oligoadenylate synthetase 1
OASL	2'-5'-oligoadenylate synthetase like
OGFR	opioid growth factor receptor
PARP12	poly(ADP-ribose) polymerase family member 12
PARP14	poly(ADP-ribose) polymerase family member 14
PARP9	poly(ADP-ribose) polymerase family member 9
PLSCR1	phospholipid scramblase 1
PNPT1	polyribonucleotide nucleotidyltransferase 1
HELZ2	helicase with zinc finger 2
PROCR	protein C receptor
PSMA3	proteasome 20S subunit alpha 3
PSMB8	proteasome 20S subunit beta 8
PSMB9	proteasome 20S subunit beta 9
PSME1	proteasome activator subunit 1

PSME2	proteasome activator subunit 2
RIPK2	receptor interacting serine/threonine kinase 2
RNF31	ring finger protein 31
RSAD2	radical S-adenosyl methionine domain containing 2
RTP4	receptor transporter protein 4
SAMD9	sterile alpha motif domain containing 9
SAMD9L	sterile alpha motif domain containing 9 like
SELL	selectin L
SLC25A28	solute carrier family 25 member 28
SP110	SP110 nuclear body protein
STAT2	signal transducer and activator of transcription 2
TAP1	transporter 1, ATP binding cassette subfamily B member
TDRD7	tudor domain containing 7
TMEM140	transmembrane protein 140
TRAFD1	TRAF-type zinc finger domain containing 1
TRIM14	tripartite motif containing 14
TRIM21	tripartite motif containing 21
TRIM25	tripartite motif containing 25
TRIM26	tripartite motif containing 26
TRIM5	tripartite motif containing 5
TXNIP	thioredoxin interacting protein
UBA7	ubiquitin like modifier activating enzyme 7
UBE2L6	ubiquitin conjugating enzyme E2 L6
USP18	ubiquitin specific peptidase 18
WARS1	tryptophanyl-tRNA synthetase 1

HALLMARK_INFLAMMATORY_GAMMA_RESPONSE: Genes up-regulated in response to IFN gamma

Gene Symbol	Gene Description
ADAR	adenosine deaminase RNA specific
APOL6	apolipoprotein L6
ARID5B	AT-rich interaction domain 5B
ARL4A	ADP ribosylation factor like GTPase 4A
AUTS2	activator of transcription and developmental regulator AUTS2
B2M	beta-2-microglobulin
BANK1	B cell scaffold protein with ankyrin repeats 1
BATF2	basic leucine zipper ATF-like transcription factor 2
BPGM	bisphosphoglycerate mutase
BST2	bone marrow stromal cell antigen 2
BTG1	BTG anti-proliferation factor 1
C1R	complement C1r
C1S	complement C1s
CASP1	caspase 1
CASP3	caspase 3
CASP4	caspase 4
CASP7	caspase 7
CASP8	caspase 8
CCL2	C-C motif chemokine ligand 2
CCL5	C-C motif chemokine ligand 5
CCL7	C-C motif chemokine ligand 7
CD274	CD274 molecule
CD38	CD38 molecule
CD40	CD40 molecule
CD69	CD69 molecule
CD74	CD74 molecule
CD86	CD86 molecule
CDKN1A	cyclin dependent kinase inhibitor 1A
CFB	complement factor B
CFH	complement factor H
CIITA	class II major histocompatibility complex transactivator
CMKLR1	chemerin chemokine-like receptor 1
CMPK2	cytidine/uridine monophosphate kinase 2
CSF2RB	colony stimulating factor 2 receptor subunit beta
CXCL10	C-X-C motif chemokine ligand 10
CXCL11	C-X-C motif chemokine ligand 11
CXCL9	C-X-C motif chemokine ligand 9
DDX58	DExD/H-box helicase 58

DDX60	DExD/H-box helicase 60
DHX58	DExH-box helicase 58
EIF2AK2	eukaryotic translation initiation factor 2 alpha kinase 2
EIF4E3	eukaryotic translation initiation factor 4E family member 3
EPSTI1	epithelial stromal interaction 1
FAS	Fas cell surface death receptor
FCGR1A	Fc fragment of IgG receptor Ia
FGL2	fibrinogen like 2
FPR1	formyl peptide receptor 1
CMTR1	cap methyltransferase 1
GBP4	guanylate binding protein 4
GBP6	guanylate binding protein family member 6
GCH1	GTP cyclohydrolase 1
GPR18	G protein-coupled receptor 18
GZMA	granzyme A
HERC6	HECT and RLD domain containing E3 ubiquitin protein ligase family member 6
HIF1A	hypoxia inducible factor 1 subunit alpha
HLA-A	major histocompatibility complex, class I, A
HLA-B	major histocompatibility complex, class I, B
HLA-DMA	major histocompatibility complex, class II, DM alpha
HLA-DQA1	major histocompatibility complex, class II, DQ alpha 1
HLA-DRB1	major histocompatibility complex, class II, DR beta 1
HLA-G	major histocompatibility complex, class I, G
ICAM1	intercellular adhesion molecule 1
IDO1	indoleamine 2,3-dioxygenase 1
IFI27	interferon alpha inducible protein 27
IFI30	IFI30 lysosomal thiol reductase
IFI35	interferon induced protein 35
IFI44	interferon induced protein 44
IFI44L	interferon induced protein 44 like
IFIH1	interferon induced with helicase C domain 1
IFIT1	interferon induced protein with tetratricopeptide repeats 1
IFIT2	interferon induced protein with tetratricopeptide repeats 2
IFIT3	interferon induced protein with tetratricopeptide repeats 3
IFITM2	interferon induced transmembrane protein 2
IFITM3	interferon induced transmembrane protein 3
IFNAR2	interferon alpha and beta receptor subunit 2
IL10RA	interleukin 10 receptor subunit alpha
IL15	interleukin 15
IL15RA	interleukin 15 receptor subunit alpha
IL18BP	interleukin 18 binding protein
IL2RB	interleukin 2 receptor subunit beta

IL4R	interleukin 4 receptor
IL6	interleukin 6
IL7	interleukin 7
IRF1	interferon regulatory factor 1
IRF2	interferon regulatory factor 2
IRF4	interferon regulatory factor 4
IRF5	interferon regulatory factor 5
IRF7	interferon regulatory factor 7
IRF8	interferon regulatory factor 8
IRF9	interferon regulatory factor 9
ISG15	ISG15 ubiquitin like modifier
ISG20	interferon stimulated exonuclease gene 20
ISOC1	isochorismatase domain containing 1
ITGB7	integrin subunit beta 7
JAK2	Janus kinase 2
KLRK1	killer cell lectin like receptor K1
LAP3	leucine aminopeptidase 3
LATS2	large tumor suppressor kinase 2
LCP2	lymphocyte cytosolic protein 2
LGALS3BP	galectin 3 binding protein
LY6E	lymphocyte antigen 6 family member E
LYSMD2	LysM domain containing 2
MARCHF1	membrane associated ring-CH-type finger 1
METTL7B	methyltransferase like 7B
MT2A	metallothionein 2A
MTHFD2	methylenetetrahydrofolate dehydrogenase (NADP+dependent)2, methenyltetrahydrofolate cyclophydrolase
MVP	major vault protein
MX1	MX dynamin like GTPase 1
MX2	MX dynamin like GTPase 2
MYD88	MYD88 innate immune signal transduction adaptor
NAMPT	nicotinamide phosphoribosyltransferase
NCOA3	nuclear receptor coactivator 3
NFKB1	nuclear factor kappa B subunit 1
NFKBIA	NFKB inhibitor alpha
NLRC5	NLR family CARD domain containing 5
NMI	N-myc and STAT interactor
NOD1	nucleotide binding oligomerization domain containing 1
NUP93	nucleoporin 93
OAS2	2'-5'-oligoadenylate synthetase 2
OAS3	2'-5'-oligoadenylate synthetase 3
OASL	2'-5'-oligoadenylate synthetase like
OGFR	opioid growth factor receptor
P2RY14	purinergic receptor P2Y14

PARP12	poly(ADP-ribose) polymerase family member 12
PARP14	poly(ADP-ribose) polymerase family member 14
PDE4B	phosphodiesterase 4B
PELI1	pellino E3 ubiquitin protein ligase 1
PFKP	phosphofructokinase, platelet
PIM1	Pim-1 proto-oncogene, serine/threonine kinase
PLA2G4A	phospholipase A2 group IVA
PLSCR1	phospholipid scramblase 1
PML	PML nuclear body scaffold
PNP	purine nucleoside phosphorylase
PNPT1	polyribonucleotide nucleotidyltransferase 1
HELZ2	helicase with zinc finger 2
PSMA2	proteasome 20S subunit alpha 2
PSMA3	proteasome 20S subunit alpha 3
PSMB10	proteasome 20S subunit beta 10
PSMB2	proteasome 20S subunit beta 2
PSMB8	proteasome 20S subunit beta 8
PSMB9	proteasome 20S subunit beta 9
PSME1	proteasome activator subunit 1
PSME2	proteasome activator subunit 2
PTGS2	prostaglandin-endoperoxide synthase 2
PTPN1	protein tyrosine phosphatase non-receptor type 1
PTPN2	protein tyrosine phosphatase non-receptor type 2
PTPN6	protein tyrosine phosphatase non-receptor type 6
RAPGEF6	Rap guanine nucleotide exchange factor 6
RBCK1	RANBP2-type and C3HC4-type zinc finger containing 1
RIPK1	receptor interacting serine/threonine kinase 1
RIPK2	receptor interacting serine/threonine kinase 2
RNF213	ring finger protein 213
RNF31	ring finger protein 31
RSAD2	radical S-adenosyl methionine domain containing 2
RTP4	receptor transporter protein 4
SAMD9L	sterile alpha motif domain containing 9 like
SAMHD1	SAM and HD domain containing deoxynucleoside triphosphate triphosphohydrolase 1
SECTM1	secreted and transmembrane 1
SELP	selectin P
SERPING1	serpin family G member 1
SLAMF7	SLAM family member 7
SLC25A28	solute carrier family 25 member 28
SOCS1	suppressor of cytokine signaling 1
SOCS3	suppressor of cytokine signaling 3
SOD2	superoxide dismutase 2
SP110	SP110 nuclear body protein

SPPL2A	signal peptide peptidase like 2A
SRI	sorcin
SSPN	sarcospan
ST3GAL5	ST3 beta-galactoside alpha-2,3-sialyltransferase 5
ST8SIA4	ST8 alpha-N-acetyl-neuraminide alpha-2,8-sialtransferase 4
STAT1	signal transducer and activator of transcription 1
STAT2	signal transducer and activator of transcription 2
STAT3	signal transducer and activator of transcription 3
STAT4	signal transducer and activator of transcription 4
TAP1	transporter 1, ATP binding cassette subfamily B member
TAPBP	TAP binding protein
TDRD7	tudor domain containing 7
TNFAIP2	TNF alpha induced protein 2
TNFAIP3	TNF alpha induced protein 3
TNFAIP6	TNF alpha induced protein 6
TNFSF10	TNF superfamily member 10
TOR1B	torsin family 1 member B
TRAFD1	TRAF-type zinc finger domain containing 1
TRIM14	tripartite motif containing 14
TRIM21	tripartite motif containing 21
TRIM25	tripartite motif containing 25
TRIM26	tripartite motif containing 26
TXNIP	thioredoxin interacting protein
UBE2L6	ubiquitin conjugating enzyme E2 L6
UPP1	uridine phosphorylase 1
USP18	ubiquitin specific peptidase 18
VAMP5	vesicle associated membrane protein 5
VAMP8	vesicle associated membrane protein 8
VCAM1	vascular cell adhesion molecule 1
WARS1	tryptophanyl-tRNA synthetase 1
XAF1	XIAP associated factor 1
XCL1	X-C motif chemokine ligand 1
ZBP1	Z-DNA binding protein 1
ZNFX1	zinc finger NFX1-type containing 1

HALLMARK_G2M_CHECKPOINT: Genes involved in the G2/M checkpoint, as in progression through the cell division cycle

Gene Symbol	Gene Description
ABL1	ABL proto-oncogene 1, non-receptor tyrosine kinase
AMD1	adenosylmethionine decarboxylase 1
ARID4A	AT-rich interaction domain 4A
ATF5	activating transcription factor 5
ATRX	ATRX chromatin remodeler
AURKA	aurora kinase A
AURKB	aurora kinase B
BARD1	BRCA1 associated RING domain 1
BCL3	BCL3 transcription coactivator
BIRC5	baculoviral IAP repeat containing 5
BRCA2	BRCA2 DNA repair associated
BUB1	BUB1 mitotic checkpoint serine/threonine kinase
BUB3	BUB3 mitotic checkpoint protein
KNL1	kinetochore scaffold 1
CASP8AP2	caspase 8 associated protein 2
CBX1	chromobox 1
CCNA2	cyclin A2
CCNB2	cyclin B2
CCND1	cyclin D1
CCNF	cyclin F
CCNT1	cyclin T1
CDC20	cell division cycle 20
CDC25A	cell division cycle 25A
CDC25B	cell division cycle 25B
CDC27	cell division cycle 27
CDC45	cell division cycle 45
CDC6	cell division cycle 6
CDC7	cell division cycle 7
CDK1	cyclin dependent kinase 1
CDK4	cyclin dependent kinase 4
CDKN1B	cyclin dependent kinase inhibitor 1B
CDKN2C	cyclin dependent kinase inhibitor 2C
CDKN3	cyclin dependent kinase inhibitor 3
CENPA	centromere protein A
CENPE	centromere protein E
CENPF	centromere protein F
CHAF1A	chromatin assembly factor 1 subunit A
CHEK1	checkpoint kinase 1

CHMP1A	charged multivesicular body protein 1A
CKS1B	CDC28 protein kinase regulatory subunit 1B
CKS2	CDC28 protein kinase regulatory subunit 2
CTCF	CCCTC-binding factor
CUL1	cullin 1
CUL3	cullin 3
CUL4A	cullin 4A
CUL5	cullin 5
DBF4	DBF4 zinc finger
DDX39A	DExD-box helicase 39A
DKC1	dyskerin pseudouridine synthase 1
DMD	dystrophin
DR1	down-regulator of transcription 1
DTYMK	deoxythymidylate kinase
E2F1	E2F transcription factor 1
E2F2	E2F transcription factor 2
E2F3	E2F transcription factor 3
E2F4	E2F transcription factor 4
EFNA5	ephrin A5
EGF	epidermal growth factor
ESPL1	extra spindle pole bodies like 1, separase
EWSR1	EWS RNA binding protein 1
EXO1	exonuclease 1
EZH2	enhancer of zeste 2 polycomb repressive complex 2 subunit
FANCC	FA complementation group C
FBXO5	F-box protein 5
FOXN3	forkhead box N3
G3BP1	G3BP stress granule assembly factor 1
GINS2	GINS complex subunit 2
GSPT1	G1 to S phase transition 1
H2AZ2	H2A.Z variant histone 2
H2AX	H2A.X variant histone
H2AZ1	H2A.Z variant histone 1
HIF1A	hypoxia inducible factor 1 subunit alpha
HIRA	histone cell cycle regulator
H2BC12	H2B clustered histone 12
HMGA1	high mobility group AT-hook 1
HMGB3	high mobility group box 3
HMGN2	high mobility group nucleosomal binding domain 2
HMMR	hyaluronan mediated motility receptor
JPT1	Jupiter microtubule associated homolog 1
HNRNPD	heterogeneous nuclear ribonucleoprotein D
HNRNPU	heterogeneous nuclear ribonucleoprotein U

HOXC10	homeobox C10
HSPA8	heat shock protein family A (Hsp70) member 8
HUS1	HUS1 checkpoint clamp component
ILF3	interleukin enhancer binding factor 3
INCENP	inner centromere protein
KATNA1	katanin catalytic subunit A1
KIF11	kinesin family member 11
KIF15	kinesin family member 15
KIF20B	kinesin family member 20B
KIF22	kinesin family member 22
KIF23	kinesin family member 23
KIF2C	kinesin family member 2C
KIF4A	kinesin family member 4A
KIF5B	kinesin family member 5B
KPNA2	karyopherin subunit alpha 2
KPNB1	karyopherin subunit beta 1
LBR	lamin B receptor
LIG3	DNA ligase 3
LMNB1	lamin B1
MAD2L1	mitotic arrest deficient 2 like 1
MAPK14	mitogen-activated protein kinase 14
MARCKS	myristoylated alanine rich protein kinase C substrate
MCM2	minichromosome maintenance complex component 2
MCM3	minichromosome maintenance complex component 3
MCM5	minichromosome maintenance complex component 5
MCM6	minichromosome maintenance complex component 6
MEIS1	Meis homeobox 1
MEIS2	Meis homeobox 2
MKI67	marker of proliferation Ki-67
MNAT1	MNAT1 component of CDK activating kinase
MT2A	metallothionein 2A
MTF2	metal response element binding transcription factor 2
MYBL2	MYB proto-oncogene like 2
MYC	MYC proto-oncogene, bHLH transcription factor
NASP	nuclear autoantigenic sperm protein
NCL	nucleolin
NDC80	NDC80 kinetochore complex component
NEK2	NIMA related kinase 2
NOLC1	nucleolar and coiled-body phosphoprotein 1
NOTCH2	notch receptor 2
NUMA1	nuclear mitotic apparatus protein 1
NUP50	nucleoporin 50
NUP98	nucleoporin 98 and 96 precursor

NUSAP1	nucleolar and spindle associated protein 1
ODC1	ornithine decarboxylase 1
ODF2	outer dense fiber of sperm tails 2
ORC5	origin recognition complex subunit 5
ORC6	origin recognition complex subunit 6
PAFAH1B1	platelet activating factor acetylhydrolase 1b regulatory subunit 1
TENT4A	terminal nucleotidyltransferase 4A
PBK	PDZ binding kinase
PDS5B	PDS5 cohesin associated factor B
PLK1	polo like kinase 1
PLK4	polo like kinase 4
PML	PML nuclear body scaffold
POLA2	DNA polymerase alpha 2, accessory subunit
POLE	DNA polymerase epsilon, catalytic subunit
POLQ	DNA polymerase theta
PRC1	protein regulator of cytokinesis 1
PRIM2	DNA primase subunit 2
PRMT5	protein arginine methyltransferase 5
PRPF4B	pre-mRNA processing factor 4B
PTTG1	PTTG1 regulator of sister chromatid separatin, securin
PTTG3P	pituitary tumor-transforming 3, pseudogene
PURA	purine rich element binding protein A
RACGAP1	Rac GTPase activating protein 1
RAD21	RAD21 cohesin complex component
RAD23B	RAD23 homolog B, nucleotide excision repair protein
RAD54L	RAD54 like
RASAL2	RAS protein activator like 2
RBL1	RB transcriptional corepressor like 1
RBM14	RNA binding motif protein 14
RPA2	replication protein A2
RPS6KA5	ribosomal protein S6 kinase A5
SAP30	Sin3A associated protein 30
KMT5A	lysine methyltransferase 5A
SFPQ	splicing factor proline and glutamine rich
SLC12A2	solute carrier family 12 member 2
SLC38A1	solute carrier family 38 member 1
SLC7A1	solute carrier family 7 member 1
SLC7A5	solute carrier family 7 member 5
SMAD3	SMAD family member 3
SMARCC1	SWI/SNF related, matrix associated, actin dependent regulator of chromatin subfamily c member 1
SMC1A	structural maintenance of chromosomes 1A
SMC2	structural maintenance of chromosomes 2
SMC4	structural maintenance of chromosomes 4

SNRPD1	small nuclear ribonucleoprotein D1 polypeptide
SQLE	squalene epoxidase
SRSF1	serine and arginine rich splicing factor 1
SRSF10	serine and arginine rich splicing factor 10
SRSF2	serine and arginine rich splicing factor 2
SS18	SS18 subunit of BAF chromatin remodeling complex
STAG1	stromal antigen 1
STIL	STIL centriolar assembly protein
STMN1	stathmin 1
SUV39H1	suppressor of variegation 3-9 homolog 1
SYNCRIP	synaptotagmin binding cytoplasmic RNA interacting protein
TACC3	transforming acidic coiled-coil containing protein 3
TFDP1	transcription factor Dp-1
TGFB1	transforming growth factor beta 1
TLE3	TLE family member 3, transcriptional corepressor
TMPO	thymopoietin
TNPO2	transportin 2
TOP1	DNA topoisomerase I
TOP2A	DNA topoisomerase II alpha
TPX2	TPX2 microtubule nucleation factor
TRA2B	transformer 2 beta homolog
TRAIP	TRAF interacting protein
TROAP	trophinin associated protein
TTK	TTK protein kinase
UBE2C	ubiquitin conjugating enzyme E2 C
UBE2S	ubiquitin conjugating enzyme E2 S
UCK2	uridine-cytidine kinase 2
UPF1	UPF1 RNA helicase and ATPase
NSD2	nuclear receptor binding SET domain protein 2
WRN	WRN RecQ like helicase
XPO1	exportin 1
YTHDC1	YTH domain containing 1
MAP3K20	mitogen-activated protein kinase kinase 20

HALLMARK_MYC_TARGETS_V2: A subgroup of genes regulated by MYC - version 2

Gene Symbol	Gene Description
AIMP2	aminoacyl tRNA synthetase complex interacting multifunctional protein 2
BYSL	bystin like
CBX3	chromobox 3
CDK4	cyclin dependent kinase 4
DCTPP1	dCTP pyrophosphatase 1
DDX18	DEAD-box helicase 18
DUSP2	dual specificity phosphatase 2
EXOSC5	exosome component 5
FARSA	phenylalanyl-tRNA synthetase subunit alpha
GNL3	G protein nucleolar 3
GRWD1	glutamate rich WD repeat containing 1
HK2	hexokinase 2
HSPD1	heat shock protein family D (Hsp60) member 1
HSPE1	heat shock protein family E (Hsp10) member 1
IMP4	IMP U3 small nucleolar ribonucleoprotein 4
IPO4	importin 4
LAS1L	LAS1 like ribosome biogenesis factor
MAP3K6	mitogen-activated protein kinase kinase kinase 6
MCM4	minichromosome maintenance complex component 4
MCM5	minichromosome maintenance complex component 5
MPHOSPH10	M-phase phosphoprotein 10
MRTO4	MRT4 homolog, ribosome maturation factor
MYBBP1A	MYB binding protein 1a
MYC	MYC proto-oncogene, bHLH transcription factor
NDUFAF4	NADH:ubiquinone oxidoreductase complex assembly factor 4
NIP7	nucleolar pre-rRNA processing protein NIP7
NOC4L	nucleolar complex associated 4 homolog
NOLC1	nucleolar and coiled-body phosphoprotein 1
NOP16	NOP16 nucleolar protein
NOP2	NOP2 nucleolar protein
NOP56	NOP56 ribonucleoprotein
NPM1	nucleophosmin 1
PA2G4	proliferation-associated 2G4
PES1	pescadillo ribosomal biogenesis factor 1
PHB	prohibitin
PLK1	polo like kinase 1
PLK4	polo like kinase 4
PPAN	peter pan homolog
PPRC1	PPARG related coactivator 1

PRMT3	protein arginine methyltransferase 3
PUS1	pseudouridine synthase 1
RABEPK	Rab9 effector protein with kelch motifs
RCL1	RNA terminal phosphate cyclase like 1
RRP12	ribosomal RNA processing 12 homolog
RRP9	ribosomal RNA processing 9, U3 small nucleolar RNA binding protein
SLC19A1	solute carrier family 19 member 1
SLC29A2	solute carrier family 29 member 2
SORD	sorbitol dehydrogenase
SRM	spermidine synthase
SUPV3L1	Suv3 like RNA helicase
TBRG4	transforming growth factor beta regulator 4
TCOF1	treacle ribosome biogenesis factor 1
TFB2M	transcription factor B2, mitochondrial
TMEM97	transmembrane protein 97
UNG	uracil DNA glycosylase
UTP20	UTP20 small subunit processome component
WDR43	WD repeat domain 43
WDR74	WD repeat domain 74

Reference

1. Aran, D.; Hu, Z.; Butte, A. J. xCell: digitally portraying the tissue cellular heterogeneity landscape. *Genome biol.* **2017**, *18*, 220.
